# Effects of endoplasmic reticulum stress on erectile function in rats with cavernous nerve injury

**DOI:** 10.1093/sexmed/qfad050

**Published:** 2023-09-03

**Authors:** Shanjie Guo, Danfeng Zhao, Zhenjie Zang, Dingchang Shao, Keqin Zhang, Qiang Fu

**Affiliations:** Department of Urology, Shandong Provincial Hospital Affiliated to Shandong First Medical University, Jinan 250021, China; Department of Urology, Shandong Provincial Hospital Affiliated to Shandong First Medical University, Jinan 250021, China; Engineering Laboratory of Urinary Organ and Functional Reconstruction of Shandong Province, Shandong Provincial Hospital Affiliated to Shandong First Medical University, Jinan 250021, China; Department of Urology, Shandong Provincial Hospital, Shandong University, Jinan 250021, China; Department of Urology, Shandong Provincial Hospital, Shandong University, Jinan 250021, China; Department of Urology, Shandong Provincial Hospital Affiliated to Shandong First Medical University, Jinan 250021, China; Engineering Laboratory of Urinary Organ and Functional Reconstruction of Shandong Province, Shandong Provincial Hospital Affiliated to Shandong First Medical University, Jinan 250021, China; Department of Urology, Shandong Provincial Hospital Affiliated to Shandong First Medical University, Jinan 250021, China; Engineering Laboratory of Urinary Organ and Functional Reconstruction of Shandong Province, Shandong Provincial Hospital Affiliated to Shandong First Medical University, Jinan 250021, China; Department of Urology, Shandong Provincial Hospital, Shandong University, Jinan 250021, China; Key Laboratory of Urinary Diseases in Universities of Shandong, Shandong First Medical University, Jinan 250021, China

**Keywords:** cavernous nerve injury, erectile dysfunction, endoplasmic reticulum stress, phenotypic modulation, smooth muscle

## Abstract

**Background:**

Erectile dysfunction (ED) occurs in an increasing number of patients after radical prostatectomy and cystectomy, and the phenotypic modulation of corpus cavernosum smooth muscle cells is closely related to ED.

**Aim:**

To determine whether endoplasmic reticulum stress (ERS) is implicated in the phenotypic modulation of ED induced by bilateral cavernous nerve injury (BCNI).

**Methods:**

In total, 36 Sprague-Dawley rats were randomly divided into 3 groups: sham, in which rats received sham surgery with bilateral cavernous nerve exposure plus phosphate-buffered saline; control, in which rats received BCNI plus phosphate-buffered saline; and experimental, in which rats received BCNI plus 4-phenylbutyric acid. Analysis of variance and a Bonferroni multiple-comparison test were utilized to evaluate differences among groups.

**Outcomes:**

Erectile function, smooth muscle/collagen ratios, and the expression levels of phenotypic modulation and ERS were measured.

**Results:**

Two ratios—maximum intracavernosal pressure/mean arterial pressure and smooth muscle/collagen—were decreased in the control group as compared with the sham group. In penile tissue, there was increased expression of GRP78 (78-kDa glucose-regulated protein), p-PERK/PERK (phosphorylated protein kinase R–like endoplasmic reticulum kinase/protein kinase R–like endoplasmic reticulum kinase), caspase 3, CHOP (C/EBP homologous protein), and OPN (osteopontin) but decreased expression of nNOS (neuronal nitric oxide synthase) and α-SMA (α–smooth muscle actin). As compared with the control group, erectile function was improved and pathologic changes were partially recovered in the experimental group.

**Clinical Translation:**

The present study demonstrated that ERS is involved in ED caused by cavernous nerve injury, thereby providing a new target and theoretical basis for clinical treatment.

**Strengths and Limitations:**

The present study demonstrated for the first time that ERS is related to ED caused by cavernous nerve injury. Inhibition of ERS reverses phenotypic modulation and improves erectile function in rats with BCNI. Additional in vitro studies should be performed to verify these conclusions and explore the specific mechanism of phenotypic modulation.

**Conclusion:**

The present study demonstrated that inhibiting ERS reverses phenotypic modulation and enhances erectile function in rats with BCNI.

## Introduction

Erectile dysfunction (ED) is defined as the inability to achieve or maintain sufficient erectile satisfaction.^1^ ED is estimated to affect 322 million men worldwide by 2025, and cavernous nerve injury (CNI)–induced ED accounts for >14% of all cases of ED.^2^^,^^3^ With the rising incidence of prostate cancer and bladder cancer, an increasing number of patients experience ED after radical prostatectomy and cystectomy. Despite the application of robotic surgery and advances in nerve repair techniques, ED caused by CNI is still an important quality-of-life problem after radical prostatectomy and cystectomy.^4^ To date, the mechanism of CNI ED has not been fully elucidated. To achieve better penile rehabilitation, many animal experiments and clinical trials have investigated new therapeutic strategies, including small molecule drugs, stem cell therapy, microenergy therapy, and platelet-rich plasma therapy.^5^^,^^6^ However, these options are often only partially effective, and more reliable interventions are urgently needed.

Previous studies found that phenotypic modulation of corpus cavernosum smooth muscle cells (CCSMCs) is a significant pathophysiologic change in ED after CNI.^7^ Smooth muscle cell (SMC) phenotypic modulation is defined as the process of transformation from a contractile phenotype to a synthetic phenotype when SMCs are stimulated by some factors.^8^^,^^9^ At present, most studies on the phenotypic modulation of corpus cavernosum smooth muscle (CCSM) are related to diabetic ED and ED caused by bilateral CNI (BCNI).^10–12^ Zhang et al noted that on the fifth day after BCNI, CCSMCs showed phenotypic transformation with increased expression of osteopontin (OPN) and decreased α–smooth muscle actin (α-SMA) and calponin.^7^ The contractile phenotype maintains an ability to contract and relax, which is important in cavernous erection. The markers of the contractile phenotype include α-SMA, calponin, and smooth muscle myosin heavy chain, while the markers of the synthetic phenotype include OPN and vimentin.^13^ However, the specific mechanism of phenotypic modulation is still unclear, which requires further study.

Furmanik et al found that endoplasmic reticulum stress (ERS) is a cellular stress response that can lead to phenotypic modulation of vascular SMCs (VSMCs).^14^^,^^15^ ERS is involved in the occurrence and development of multiple diseases, including Parkinson’s disease, atherosclerosis, autoimmune diseases, and cancer.^16^ ERS is activated when misfolded or unfolded proteins in the endoplasmic reticulum exceed the capacity of the endoplasmic reticulum, thereby activating a set of signaling pathways called the unfolded protein response (UPR).^17^ The UPR signaling pathway reduces the folding load of the endoplasmic reticulum by reducing protein synthesis and transport into the endoplasmic reticulum.^18^ If ERS is chronically prolonged, the UPR will result in cell dysfunction and apoptosis. The activation of apoptosis leads to the occurrence and progression of diseases. C/EBP homologous protein (CHOP) is one of the effectors of UPR induced by ERS, which can promote cell proliferation in VSMCs.^19^ When the expression of PERK and CHOP is high, it promotes the transformation of VSMCs from the contractile phenotype to the synthetic.^20^ This phenotype results in increased proliferation, migration, and secretion of extracellular matrix–related proteins, leading to pathologic phenotypic transformation, such as the formation of fibrous caps on atherosclerotic plaques.^9^^,^^18^ Importantly, ERS related to phenotypic modulation caused by BCNI ED has not been previously studied.

Is ERS involved in CNI ED? In this research, we explored the significant role of ERS in the phenotypic modulation of rats with ED caused by BCNI, and we demonstrated that inhibition of ERS caused the reversal of the phenotypic modulation of CCSM, thus improving erectile function.

## Methods

### Experimental design

**Figure 1:**
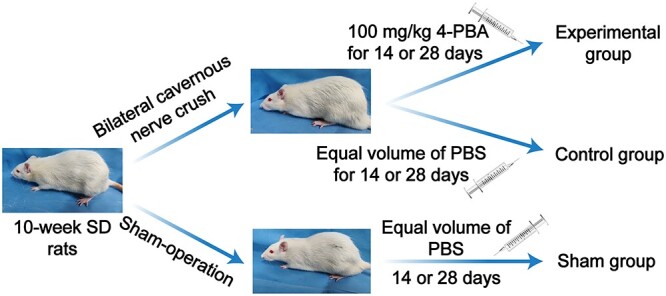
Animal models and experimental design.

The Animal Care and Use Committee of Shandong Provincial Hospital Affiliated to Shandong First Medical University approved the experimental protocols (No. 2021-049). The Shandong Provincial Hospital Laboratory Animal Center provided 36 ten-week-old male Sprague-Dawley rats weighing 320 to 350 g with normal erectile function. At the Shandong Provincial Hospital Laboratory Animal Center, the rats were kept on a 12:12 light-dark cycle with unlimited access to food and water. The 36 Sprague-Dawley rats were randomly assigned to 1 of 3 groups (n = 12 each): sham operation, control, and experimental. In the sham group, the bilateral cavernous nerves of rats were exposed without further operation. In the control and experimental groups, the bilateral cavernous nerves were compressed via a hemostatic clamp (2-mm diameter) for 1 minute.^21^ The experimental group began receiving an intraperitoneal injection of 4-phenylbutyric acid (4-PBA; 100 mg/kg/d [Selleck]) starting on day 1 postoperation.^22^ The sham and control groups received an equal volume of phosphate-buffered saline. To investigate the time changes in the cavernous body, a subgroup of 6 rats in each group was established on day 14 or 28 postoperation. At day 14 or 28, the mean arterial pressure (MAP) and maximum intracavernosal pressure (max ICP) were measured to evaluate erectile function. We exerted every effort to minimize the suffering of the experimental animals (Figure 1).

### Erectile function evaluation

At day 14 or 28 postoperation, we assessed erectile function by measuring max ICP and MAP. After anesthesia, the bilateral cavernous nerves were exposed. The right penile crus was then exposed, and a 23-gauge needle containing heparin solution was inserted for ICP measurement. One 24-gauge indwelling needle connected to a PE-50 tube containing heparin was inserted into the left carotid artery to monitor systemic MAP. The stimulus parameters of the cavernous nerve were 5 V, 15 Hz, 5-millisecond pulse width, and 60-second duration. The changes in MAP and ICP were recorded by a pressure transducer system (PowerLab26T; AD Instruments). The data were analyzed by LabChart8 software (AD Instruments). The penises were harvested for further examination.

### Masson trichrome staining

Penile tissues were fixed and prepared for histologic examination. The Masson trichrome stain was used to measure the smooth muscle/collagen content of the corpus cavernosum. In short, muscle fibers were stained red, while collagenous fibers were colored blue. The smooth muscle/collagen ratios were assessed by Image-Pro Plus 5.1 (Media Cybernetics).

### Immunofluorescence staining

The middle part of the penis was excised for immunofluorescence detection. Penile tissue was fixed with 4% paraformaldehyde and dehydrated overnight in phosphate-buffered saline containing 30% sucrose. The samples were then embedded and cut into 5-μm sections. The penile sections were incubated with the following primary antibodies: glucose regulatory protein 78 (GRP78, 1:100; Abcam), caspase 3 (1:100; Novus Biologicals), α-SMA (1:100; CST), and OPN (1:100; Novus Biologicals). The sections were then incubated with an Alexa Fluor 594–conjugated secondary antibody (Invitrogen). DAPI was used to stain the nuclei. Digital images were obtained by visualizing the signals with a confocal laser scanning microscope (Leica).

### Western blot analysis

The protein levels in the penile tissues were measured by Western blotting. After the penis tissue was taken out, it was fully ground in liquid nitrogen. It was then lysed with radioimmunoprecipitation assay lysis buffer (Thermo Fisher Scientific), and the protein concentrations were detected by a bicinchoninic acid protein detection kit. In 10% sodium dodecyl sulfate–polyacrylamide gel electrophoresis gels, equal amounts of protein were injected, electrophoresed, and transferred to polyvinylidene fluoride membranes. These membranes were blocked with 5% skimmed milk for 2 hours and incubated overnight at 4 °C with the following primary antibodies: anti–neuronal nitric oxide synthase (anti-nNOS, 1:1000; Abcam), anti-GRP78 (1:1000; Abcam), anti-CHOP (1:1000; CST), anti-PERK (1:1000; CST), anti–p-PERK (1:1000; CST), anti–caspase 3 (1:1000; Novus Biologicals), anti–α-SMA (1:1000; CST), and anti-OPN (1:1000; Novus Biologicals). After the membranes were washed 3 times, they were incubated with secondary antibody at room temperature for 1 hour. An enhanced chemiluminescence system (SH-Compact 523; SHST) was used to detect the protein bands, which were then quantitatively analyzed with ImageJ (version 1.52a; National Institutes of Health). The protein levels were normalized to the levels of β-actin or GAPDH (1:1000; CST).

### Statistical analyses

All experimental data are expressed as mean ± SD. All data were analyzed by Prism (version 8.0; GraphPad) and SPSS (version 25.0; IBM). Analysis of variance and a Bonferroni multiple-comparison test were utilized to evaluate differences between groups. The *t*-test was used to compare the differences between 2 groups. *P* < .05 was regarded as statistically significant.

## Results

### Erectile function evaluation

**Figure 2:**
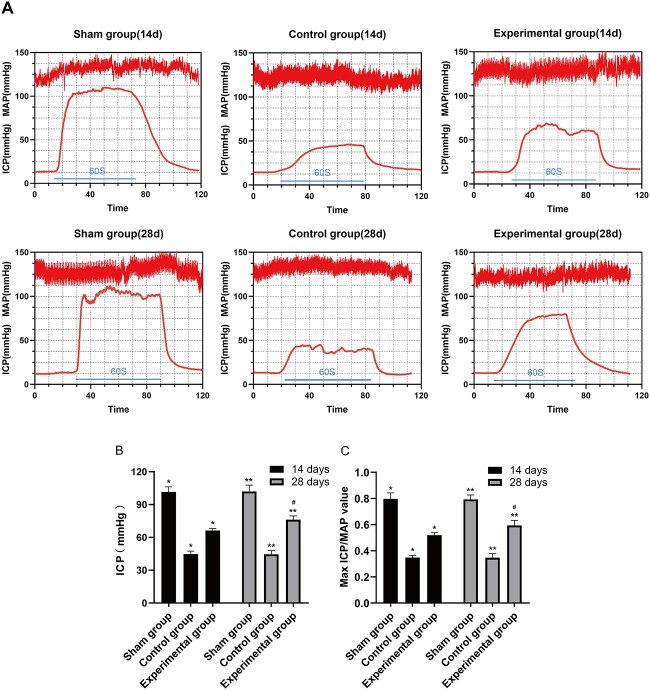
(A) ICPs of rats in different groups. The horizontal line indicates that the electrical stimulation time of the cavernous nerve was 60 seconds. (B) ICPs and (C) maximum ICP/MAP ratios. Data are shown as mean ± SD (n = 6 per group). ^*^P < .01 vs each group per ANOVA and Bonferroni multiple-comparison test. ^*^^*^P < .01 vs the group per ANOVA and Bonferroni multiple-comparison test. ^#^*P* < .05 vs the experimental group at day 14 per a *t*-test. ANOVA, analysis of variance; ICP, intracavernosal pressure; MAP, mean arterial pressure.

Erectile function was evaluated at day 14 or 28 postoperation. The sham group showed a normal cavernous body pressure curve. The max ICP/MAP ratios at day 14 or 28 in the control group (0.35 ± 0.02 and 0.35 ± 0.03, respectively) were lower than those in the sham group (0.80 ± 0.05 and 0.79 ± 0.03; all *P* < .01). The max ICP/MAP ratios in the experimental group (0.52 ± 0.02 and 0.59 ± 0.04) were increased as compared with the control group (all *P* < .01). In the experimental group, the max ICP/MAP ratio at day 28 was higher than that at day 14 (*P* < .05; Figure 2).

### 4-PBA reduces smooth muscle fibrosis

**Figure 3:**
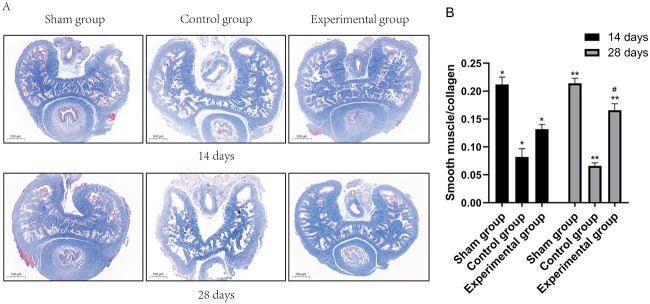
4-Phenylbutyric acid increases the smooth muscle content in bilateral cavernous nerve injury rats. (A) Penile tissues of the 3 groups were stained with Masson trichrome at day 14 or 28 after treatment. (B) Smooth muscle/collagen ratio among the groups. Data are shown as mean ± SD (n = 5 per group). ^*^P < .01 vs each group per ANOVA and Bonferroni multiple-comparison test. ^*^^*^P < .01 vs each group per ANOVA and Bonferroni multiple-comparison test. ^#^*P* < .05 vs the experimental group at day 14 per a *t*-test. ANOVA, analysis of variance.

Figure 3 shows that the smooth muscle/collagen ratios at days 14 and 28 in the control group (0.08 ± 0.01 and 0.07 ± 0.01, respectively) were lower than those in the sham group (0.21 ± 0.01 and 0.21 ± 0.01; all *P* < .01). The smooth muscle/collagen ratios in the experimental group (0.13 ± 0.01 and 0.17 ± 0.01) were higher than those in the control groups (all *P* < .01) but lower than those in the sham group (all *P* < .01). In the experimental group, the smooth muscle/collagen ratio at day 28 was higher than that at day 14 (*P* < .05).

### Effects of 4-PBA on nNOS expression

**Figure 4:**
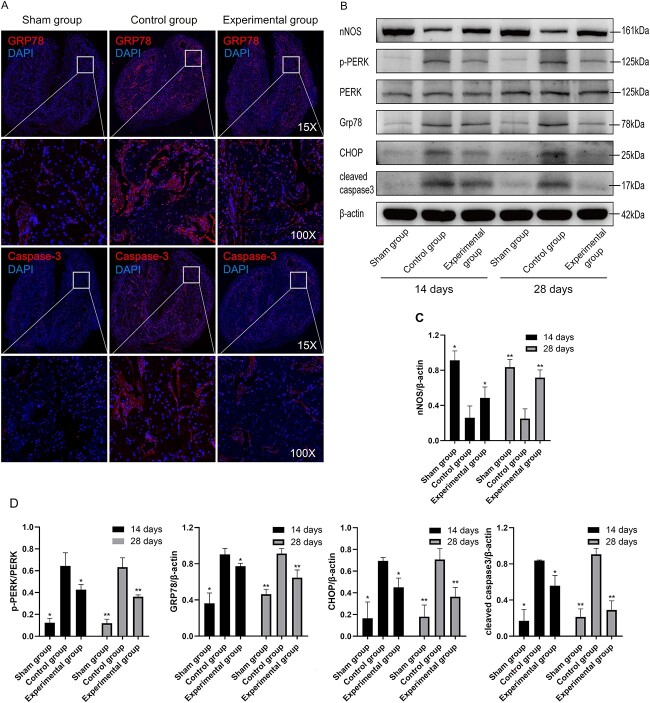
(A) Immunofluorescence staining of GRP78 and caspase 3 in cavernous tissue (day 28). (B-D) The expression levels of nNOS- and ERS-related proteins were detected by Western blot analysis. Data are reported as mean ± SD (n = 3 per group). ^*^P < .05 vs the control group at 14 days per ANOVA and Bonferroni multiple-comparison test. ^*^^*^P < .05 vs the control group at 28 days per ANOVA and Bonferroni multiple-comparison test. ANOVA, analysis of variance; ERS, endoplasmic reticulum stress; nNOS, neuronal nitric oxide synthase.

Western blotting was used to evaluate the CCSM nNOS of penile tissues in the 3 groups at day 14 or 28 postoperation. Figure 4B and C shows that nNOS expression in the control group was lower than that in the sham group (all *P* < .05). In addition, nNOS expression in the experimental group was higher than that in the control group (all *P* < .05).

### 4-PBA reduces ERS

The protein levels in penile tissue at day 14 or 28 postoperation were detected by immunofluorescence and Western blotting (Figure 4). The protein levels of GRP78, p-PERK/PERK, caspase 3, and CHOP in the control group were higher than those in the sham and experimental groups (all *P* < .05). Interestingly, the aforementioned proteins in the experimental group were lower than those in the control group (GRP78, p-PERK/PERK, caspase 3, and CHOP; all *P* < .05). Furthermore, the levels of ERS-related proteins in the experimental group were lower than those in the control group, indicating that 4-PBA inhibited ERS.

### 4-PBA increases α-SMA expression and decreases OPN expression

**Figure 5:**
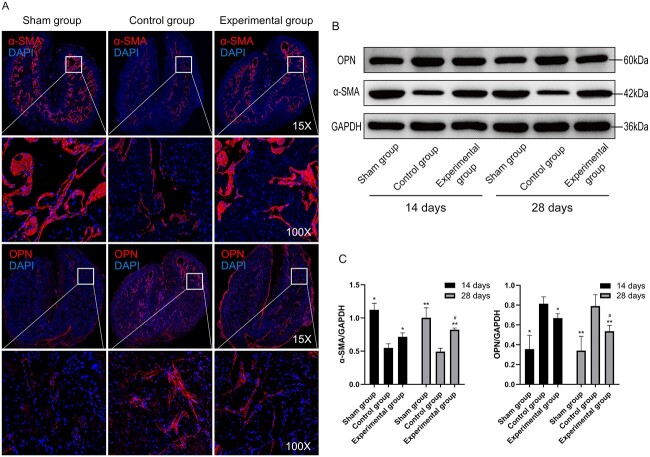
4-Phenylbutyric acid increases α-SMA expression and decreases osteopontin expression. (A) Immunofluorescence staining of α-SMA and OPN in cavernous tissue (day 28). (B-D) Western blot analysis was employed to quantitate the levels of α-SMA and OPN. Data are reported as mean ± SD (n = 3 per group). ^*^P < .05 vs the control group at 14 days per ANOVA and Bonferroni multiple-comparison test. ^*^^*^P < .05 vs the control group at 28 days per ANOVA and Bonferroni multiple-comparison test. ^#^*P* < .05 vs the experimental group at 14 days per a *t*-test. α-SMA, α–smooth muscle actin; ANOVA, analysis of variance.

The expression levels of OPN and α-SMA in rat penile tissue were measured by immunofluorescence and Western blotting (Figure 5). In the sham group, the expressions of α-SMA and OPN were normal. At day 14 or 28 postoperation, the α-SMA levels of the control group were lower than those of the sham group (all *P* < .05), while the OPN levels of the control group were higher (all *P* < .05). Interestingly, the experimental group’s α-SMA levels were higher than the control group’s (all *P* < .05), while the experimental group’s OPN levels were lower (all *P* < .05). In the experimental group, the protein level of α-SMA at day 28 was increased as compared with day 14 (*P* < .05). Moreover, the OPN protein levels at day 28 was decreased vs day 14 (*P* < .05). 4-PBA thus increases α-SMA expression and decreases OPN expression.

## Discussion

ED occurs in an increasing number of patients after radical prostatectomy and cystectomy, and it has been found that the phenotypic modulation of CCSMC is closely related to ED.^23^^,^^24^ However, the mechanism of phenotypic modulation of cavernous smooth muscle has not been fully elucidated. Therefore, it is essential to further research the mechanism of phenotypic modulation and identify a more effective and safer method for the treatment of CNI-induced ED. ERS leads to the phenotypic modulation of SMCs. Thus, we hypothesized that inhibition of ERS may reverse the phenotypic modulation to improve erectile function.

After BCNI, the phenotypic modulation of CCSM occurs in control group. Yang et al found CCSM phenotypic modulation and increased protein expression of hypoxia-inducible factor 1α and collagen in corpora cavernous tissue.^24^ Tang et al reported that the RhoA/ROCK signaling pathway plays a significant role in the phenotypic regulation of CCSMCs.^25^ miRNAs are closely related to the occurrence of diseases, and mir195-5p may regulate the phenotypic transformation of CCSM cells through Smad7.^26^ In the present research, we established a typical BCNI rat model, in which erectile function was decreased in the control group. Masson trichrome stain demonstrated that smooth muscle/collagen fibers were decreased at days 14 and 28 postoperation, while immunofluorescence and Western blotting showed that α-SMA levels decreased but OPN levels increased in the control group. We observed phenotypic modulation in BCNI rats, which agreed with the results of Yang et al and Qinyu et al.^10^ In the cardiovascular system, the phenotypic modulation of VSMCs is one of the basic pathophysiologic changes of atherosclerosis, vascular stenosis, and other diseases.^27^^,^^28^ SMCs present 2 reversible phenotypes—contractile and synthetic—with different roles for each. In general, the contractile phenotype maintains an ability to contract and relax, while the synthetic phenotype promotes proliferation, migration, inflammatory signals, calcification, and extracellular matrix production.^9^ In the present study, after CNI, the contractile phenotype of CCSM was decreased, and the synthetic phenotype was increased, which led to a decline in the contractile and relaxation ability of cavernous smooth muscle. These findings were consistent with those by Yang et al. Therefore, these findings suggest that the decrease in erectile function after BCNI may be related to the phenotypic modulation of CCSM from a contractile phenotype to a synthetic phenotype.

The level of ERS in CCSM increased after BCNI. ERS is a cellular stress response in which ERS is activated when the need for protein folding exceeds the capacity of the endoplasmic reticulum.^29^^,^^30^ In the present research, BCNI resulted in decreased erectile function and increased expression of GRP78 and p-PERK/PERK in the cavernous tissue of the control group as compared with the sham group. Previous research has indicated that GRP78 is a key protein in ERS that binds to 3 proteins in the UPR pathway that are responsible for sensing unfolded proteins: inositol demand factor 1 (IRE1), activating transcription factor 6 (ATF6), and PERK.^31^^,^^32^ When ERS occurs, GRP78 is recruited by unfolded or misfolded proteins and dissociates from IRE1, ATF6, and PERK. After dissociation, these 3 proteins are activated, further inducing the downstream UPR signal.^33^ At the same time, the levels of caspase 3 and CHOP, which are downstream proteins of GRP78 and PERK, are increased. When the intensity of continuous stress is too high, it leads to activation of the CHOP and caspase families in the downstream apoptotic pathway, eventually leading to apoptosis and disease.^16^^,^^34^ Similar results occurred in the present research. By establishing the BCNI model in rats, we found that erectile function decreased and the expression levels of GRP78, p-PERK/PERK, caspase 3, and CHOP in penile cavernous tissue increased in the control group. These findings suggest that ERS may be involved in the pathogenesis of ED caused by BCNI in rats.

ERS induces the phenotypic modulation of CCSM and may participate in BCNI-induced ED. In the present research, we found that phenotypic modulation of CCSM occurred after CNI. We utilized 4-PBA, a common ERS inhibitor, to verify the important role of ERS involved in the phenotypic switching of CCSM. When compared with the control group, rats’ erectile function was partially improved after the addition of 4-PBA, and the erectile function on day 28 was higher than that on day 14. After treatment with 4-PBA, the levels of GRP78, p-PERK/PERK, caspase 3, CHOP, and OPN were decreased, whereas the levels of α-SMA were increased. Previous research has demonstrated that ERS promotes the switching of SMCs from a contractile phenotype to a synthetic phenotype.^35^ Zhao et al reported that ERS participates in the phenotypic transformation of VSMCs through the Notch pathway,^36^ and Zhang et al determined that ERS promotes the phenotypic transformation of rat VSMCs through the PERK-eIF2a-ATF4-CHOP pathway,^20^^,^^37^ in line with the present results. In our research, we demonstrated that 4-PBA reduced the stress level of the endoplasmic reticulum and reversed the phenotypic transition from the contractile phenotype to the synthetic phenotype. Thus, these findings suggest that ERS may induce phenotypic modulation and be involved in BCNI-induced ED. Moreover, the present study indicates that inhibition of ERS could improve erectile function in CNI rats.

We verified that inhibition of ERS reversed the decrease in contractile protein levels and improved erectile function. However, there were limitations in determining the specific ERS downstream pathway, suggesting that additional in vitro experiments are required to verify the present conclusions and explore the specific mechanism of phenotypic modulation induced by ERS in BCNI rats. In addition, we used relatively few animals, and there was no research on age in this study. Our research on ED is ongoing, and we will continue to explore potential clinical application in future study.

## Conclusion

In the present study, we demonstrated that BCNI increases the protein levels of GRP78, p-PERK/PERK, caspase 3, CHOP, and OPN and decreases the protein levels of α-SMA. In addition, we showed that 4-PBA improves erectile function by inhibiting the level of ERS-related proteins, increasing the protein level of α-SMA (a smooth muscle contractile protein), and inhibiting the expression of OPN (a synthetic component protein) in BCNI rats. Moreover, inhibition of ERS reverses phenotypic modulation and improves erectile function in rats with BCNI. Thus, the present study demonstrated for the first time that ERS is involved in BCNI-induced ED, thereby providing a new target and theoretical basis for the clinical treatment of CNI-induced ED.

## Supplementary Material

WB_Supplemental_Figure1_qfad050

Body_Weight_Supplemental_Table1_qfad050
