# Supplementary material for: Effects of endoplasmic reticulum stress on erectile function in rats with cavernous nerve injury
Source: Sex Med. 2023 Sep 3;11(4):qfad050. doi: 10.1093/sexmed/qfad050 (PMC10478027; doi:10.1093/sexmed/qfad050)

WB Supplemental Figure1

Sample 1

nNOS
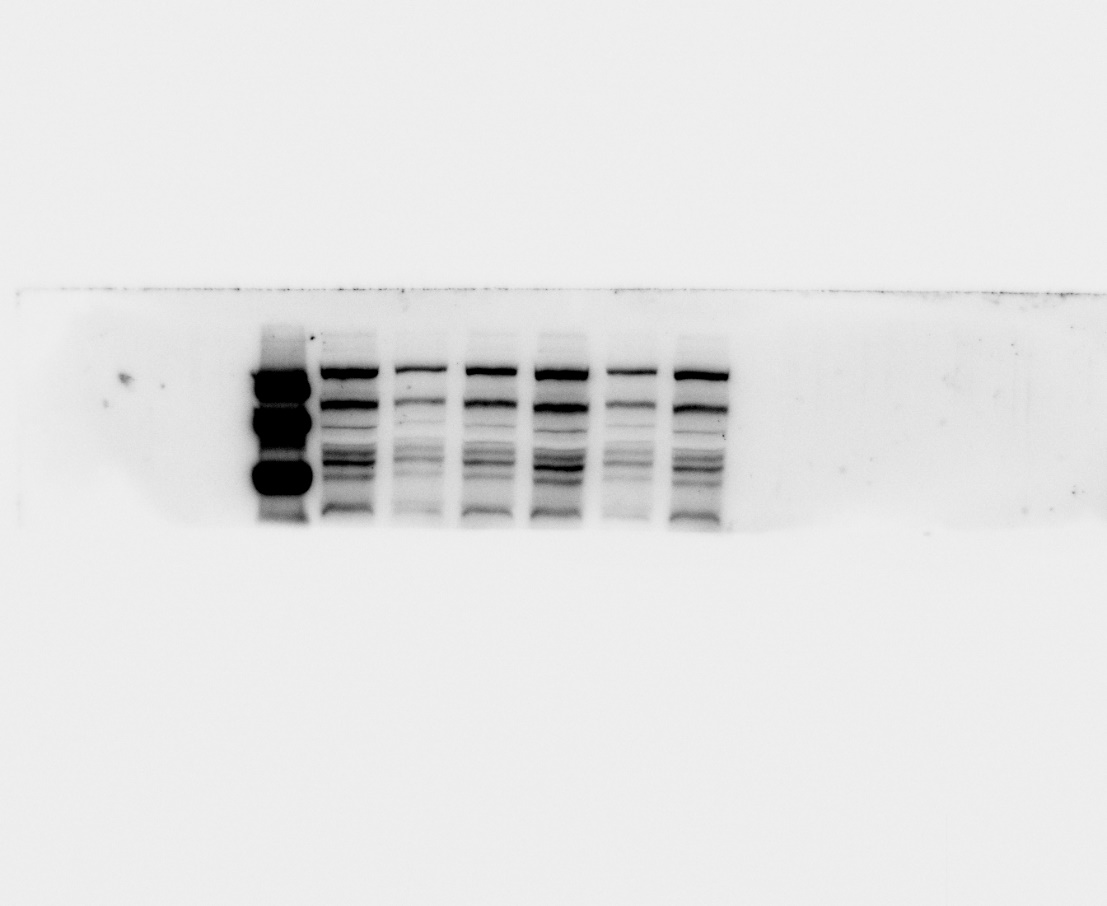


p-PERK
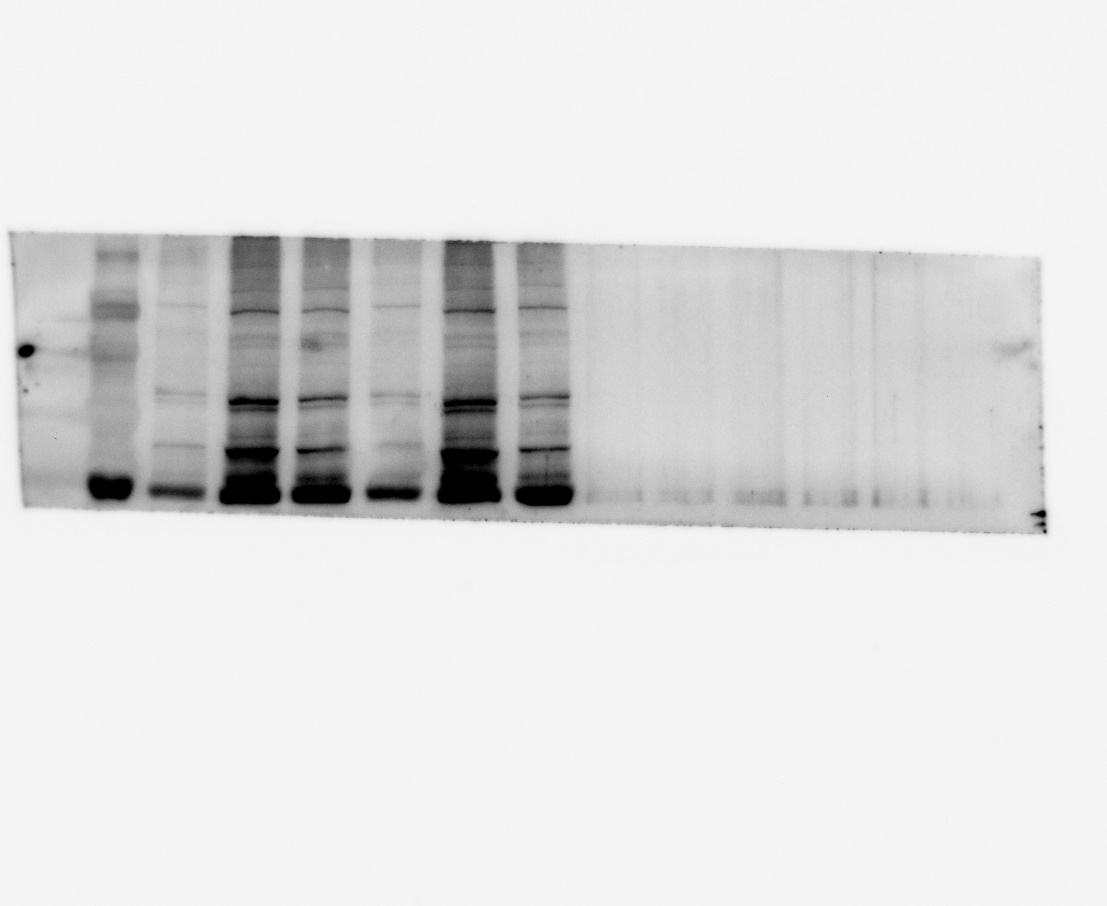


PERK
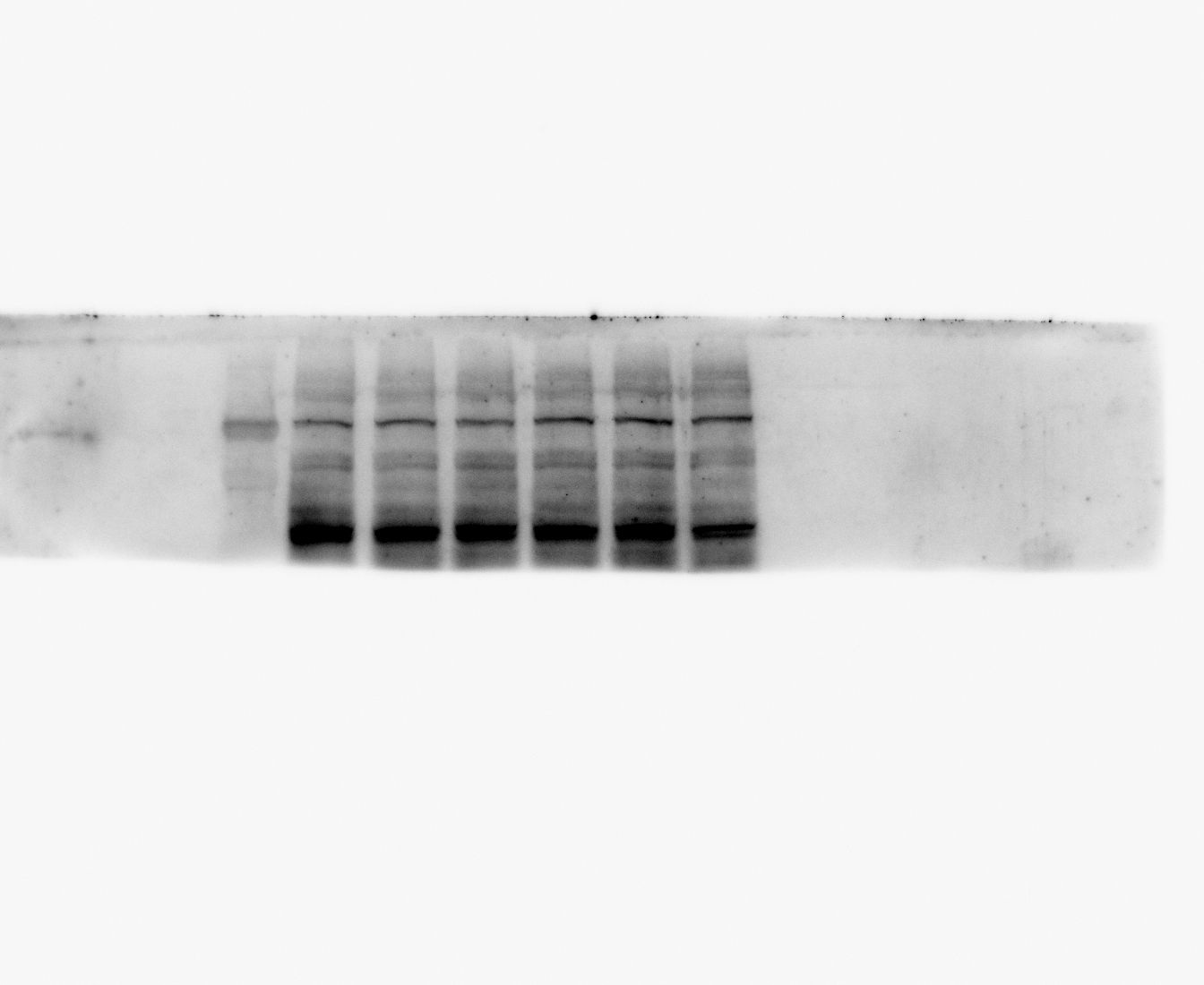


GRP78
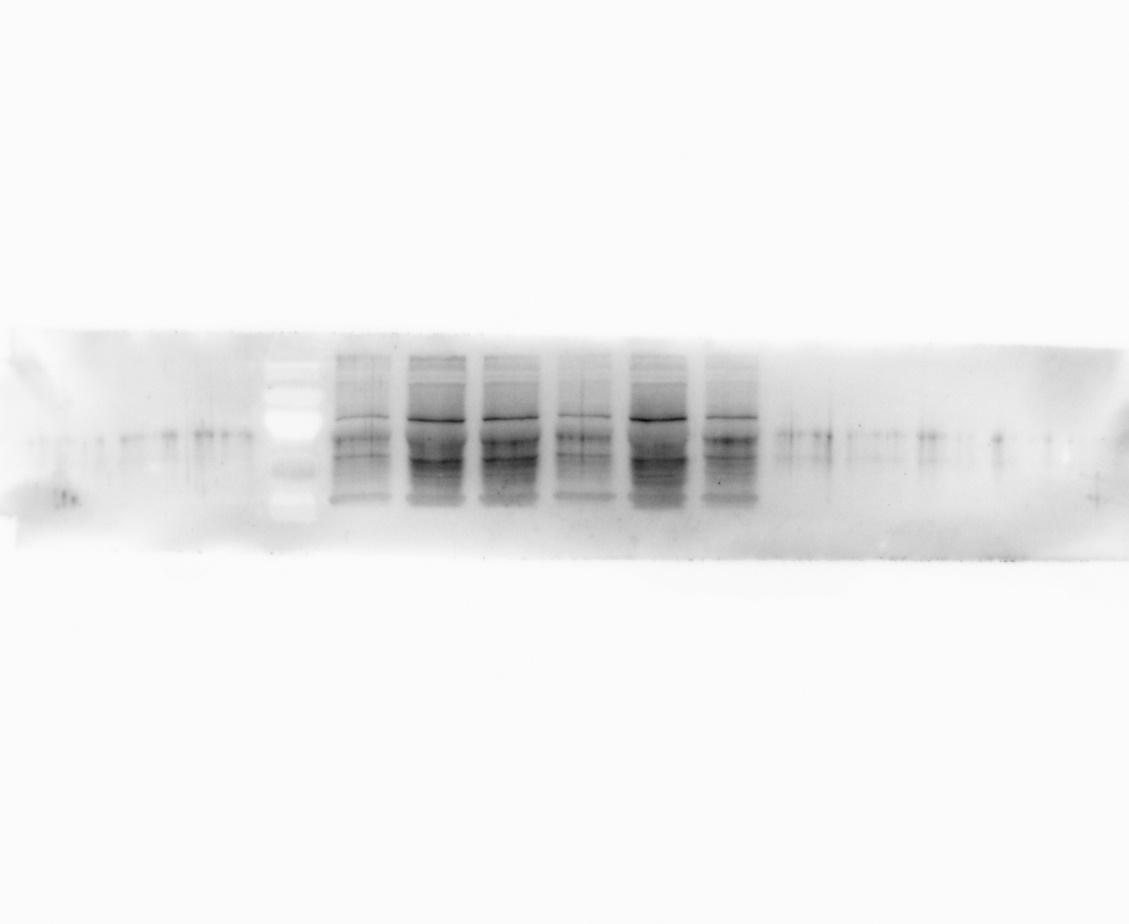


CHOP
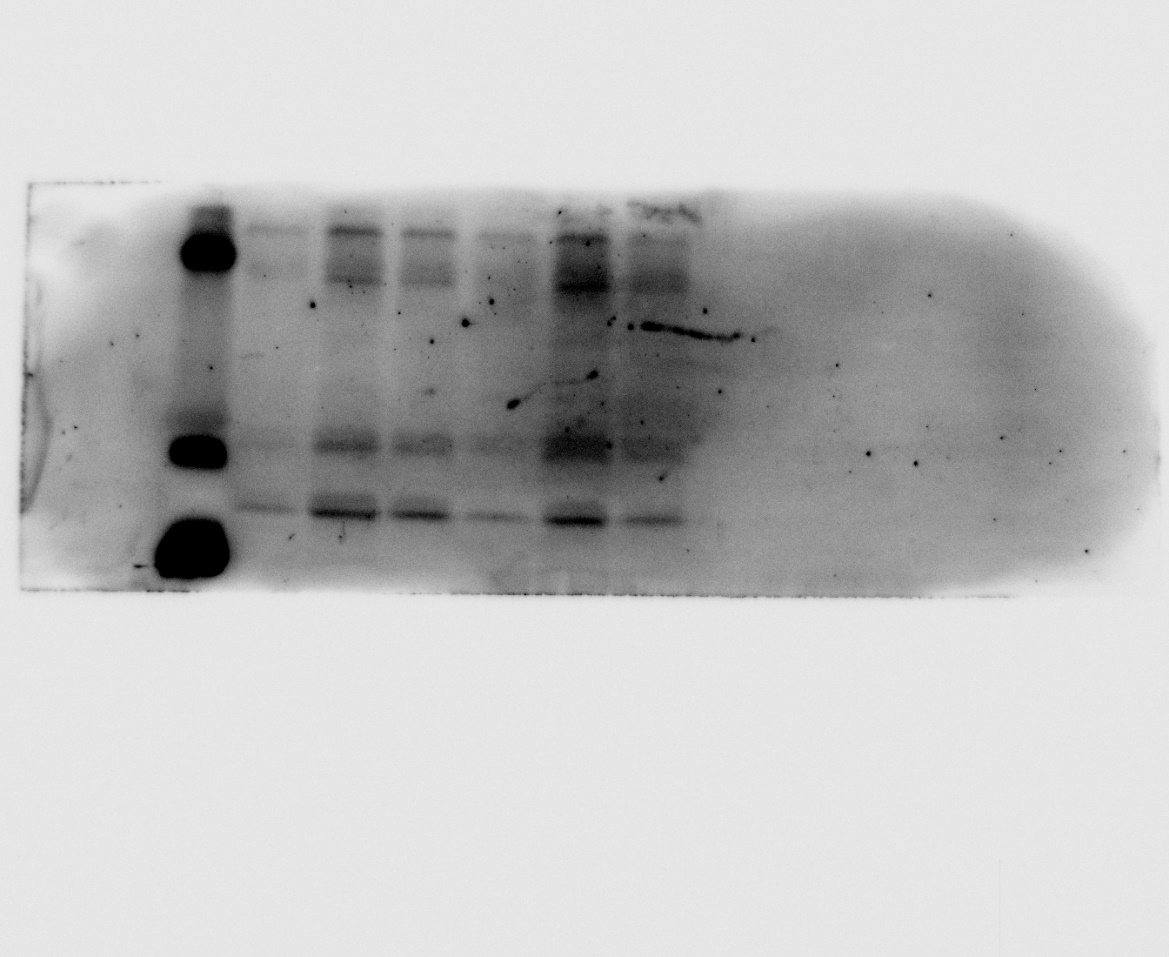


Cleaved caspase-3
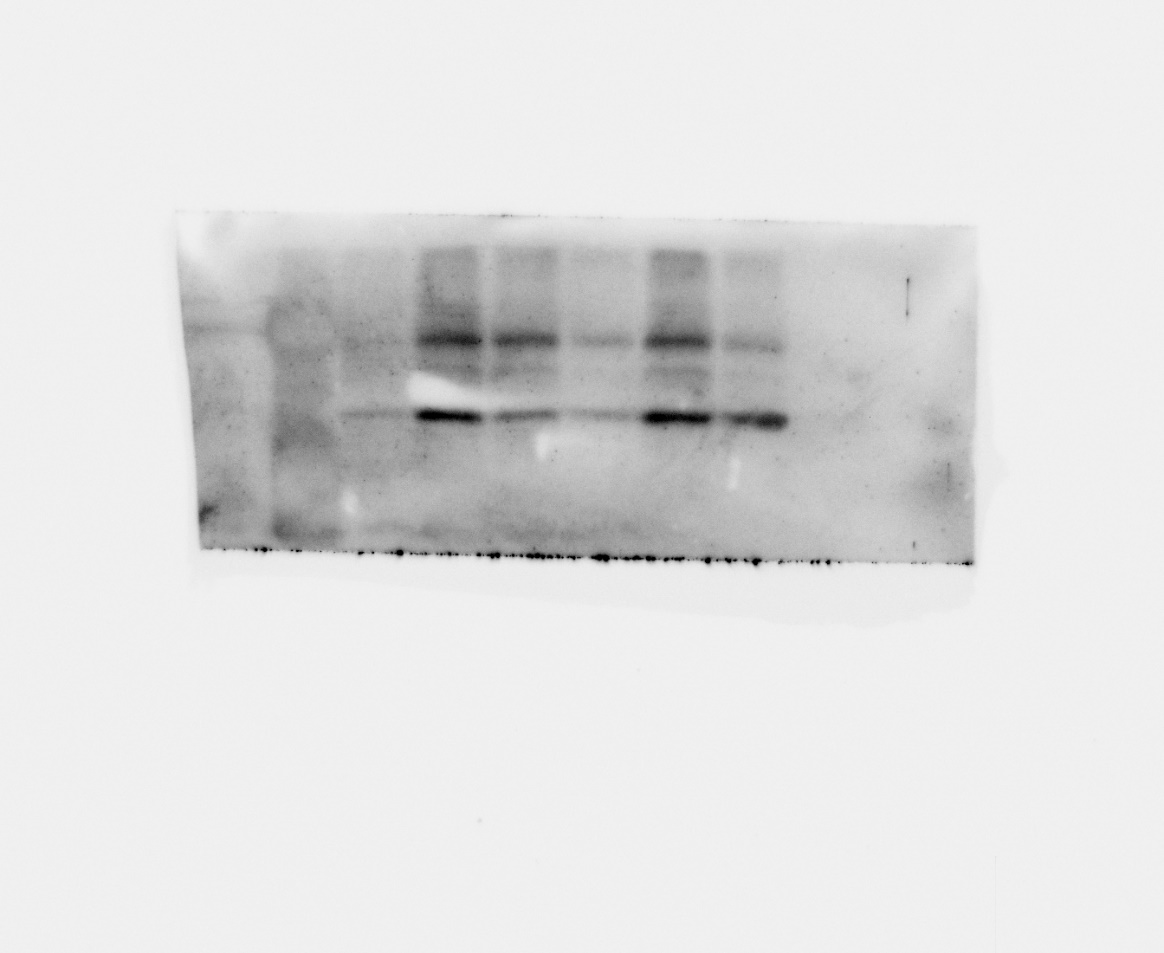


β-actin
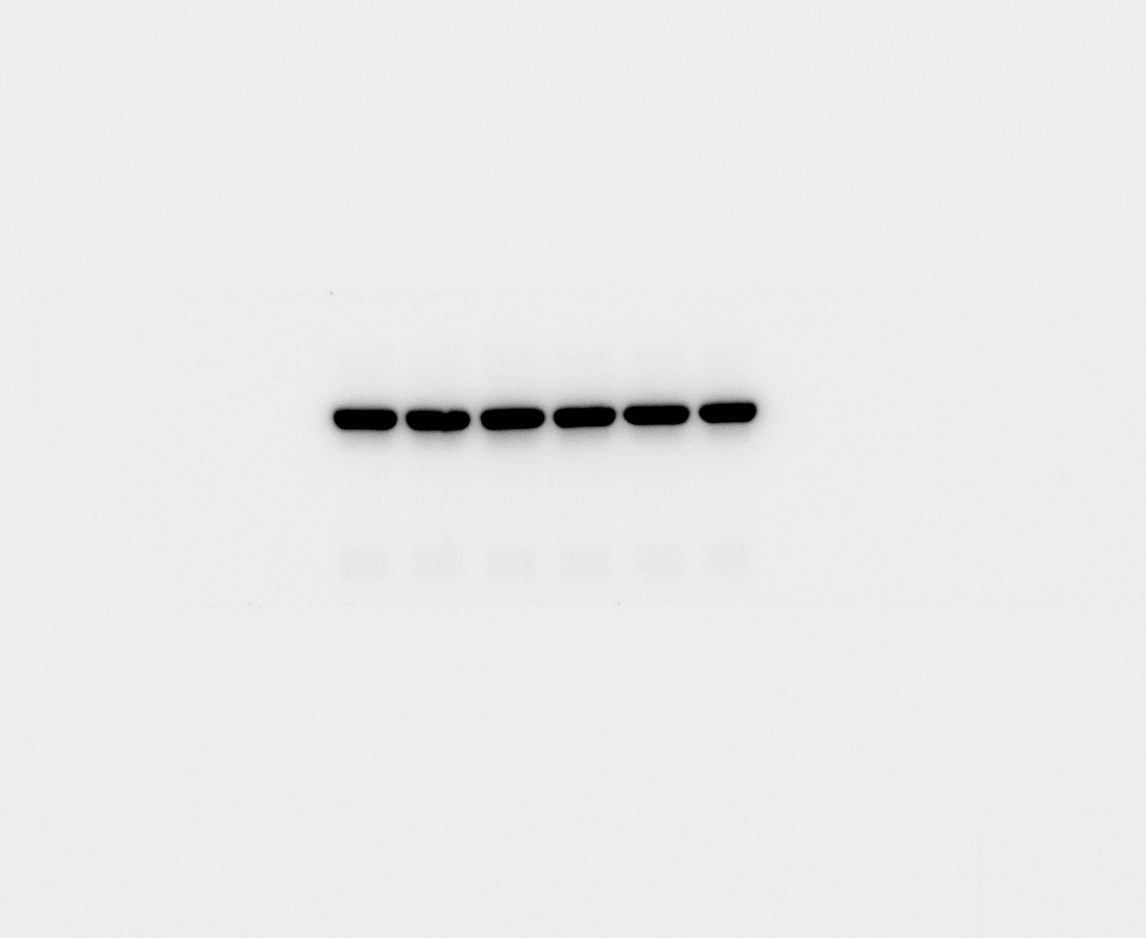


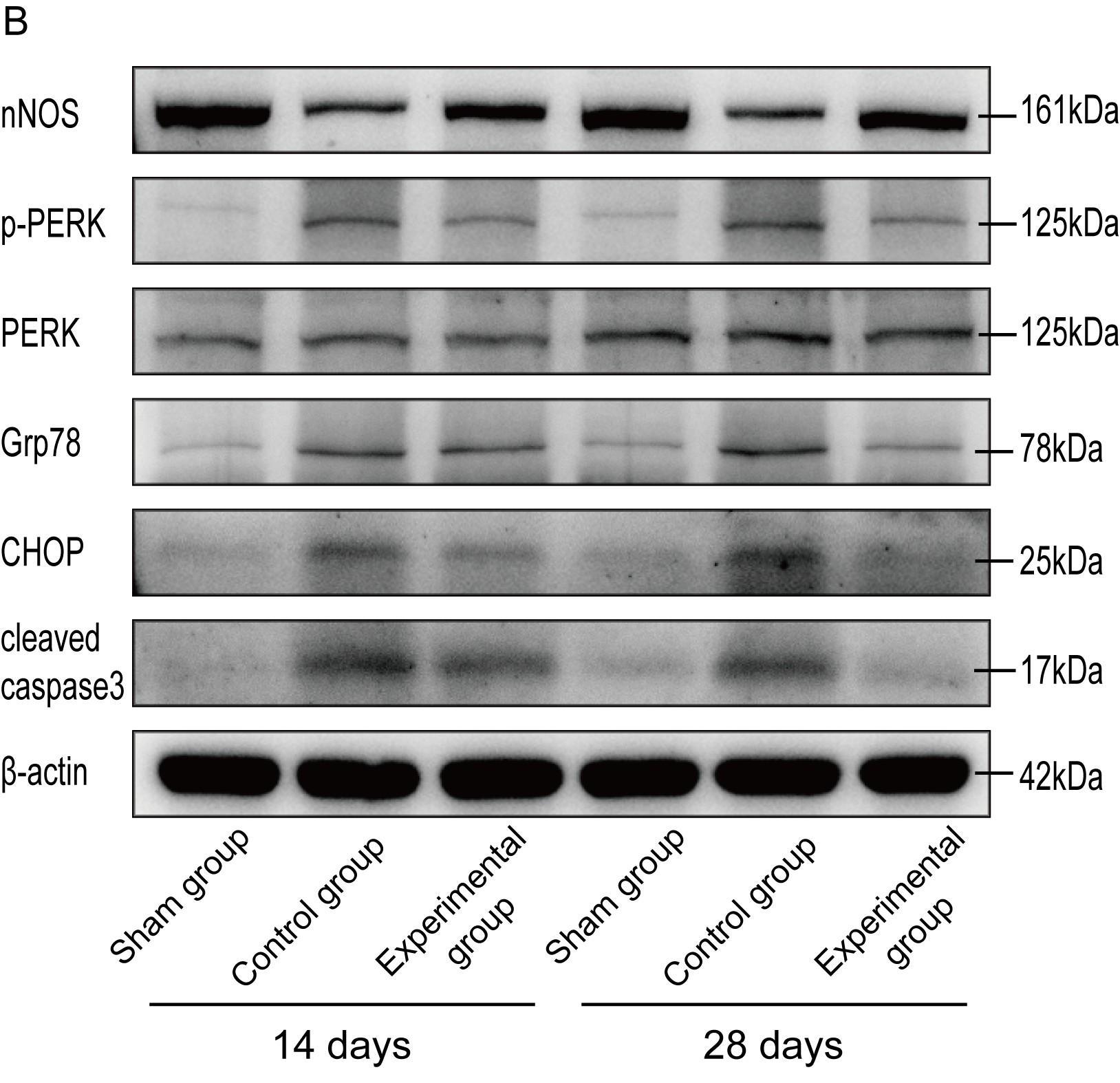


OPN
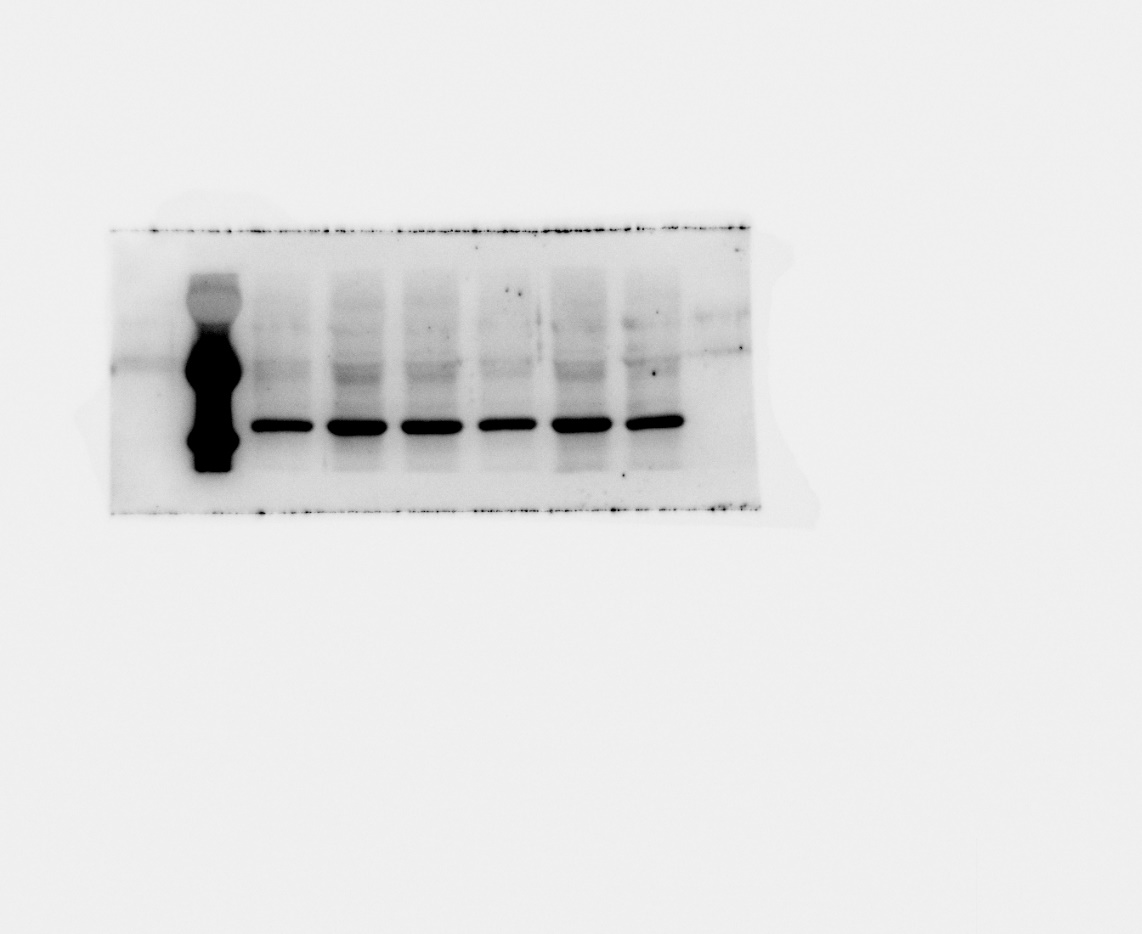


α-SMA
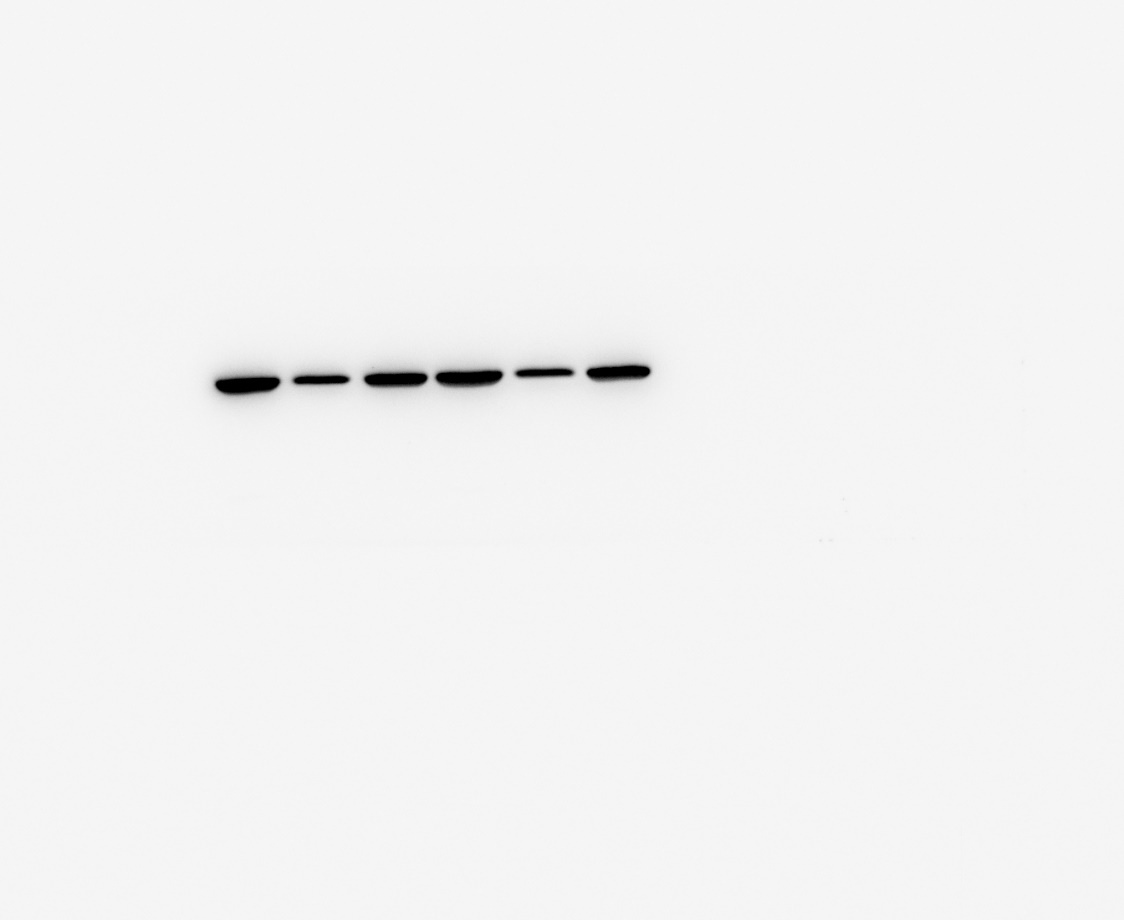


GAPDH
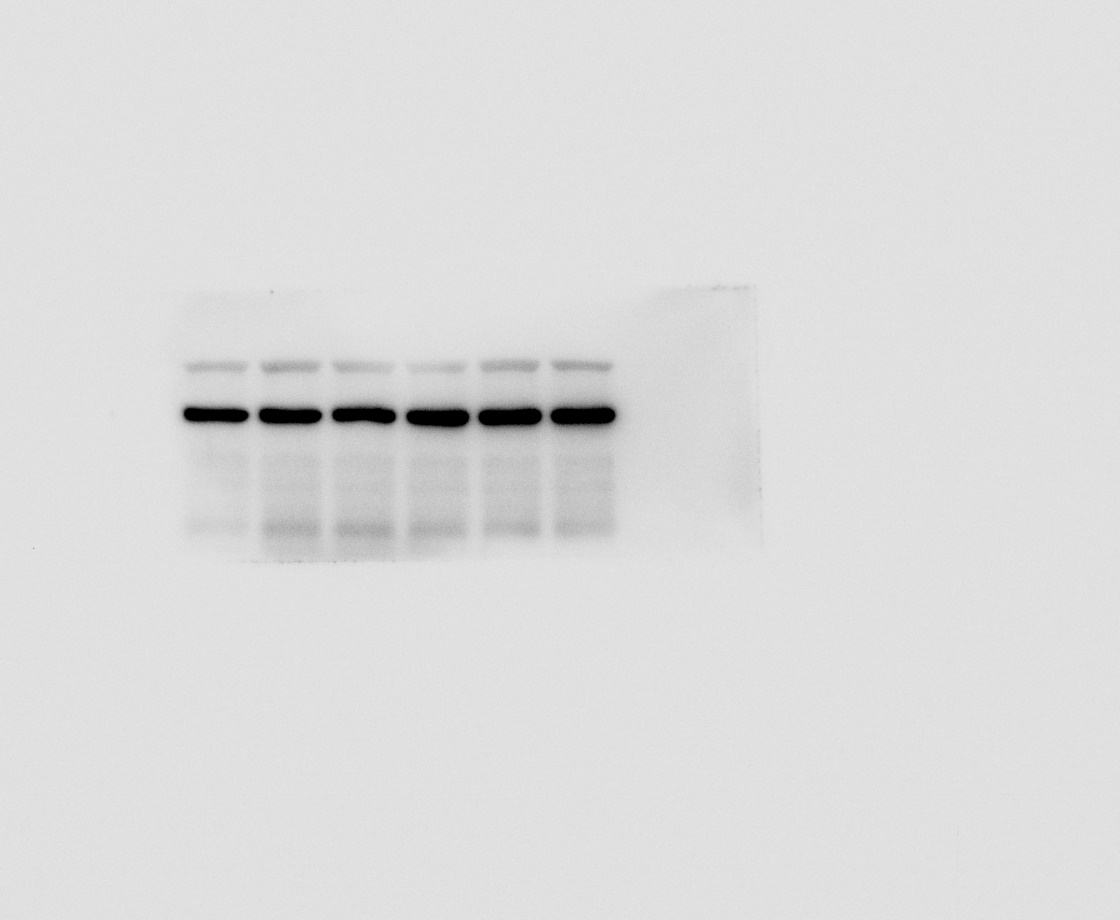


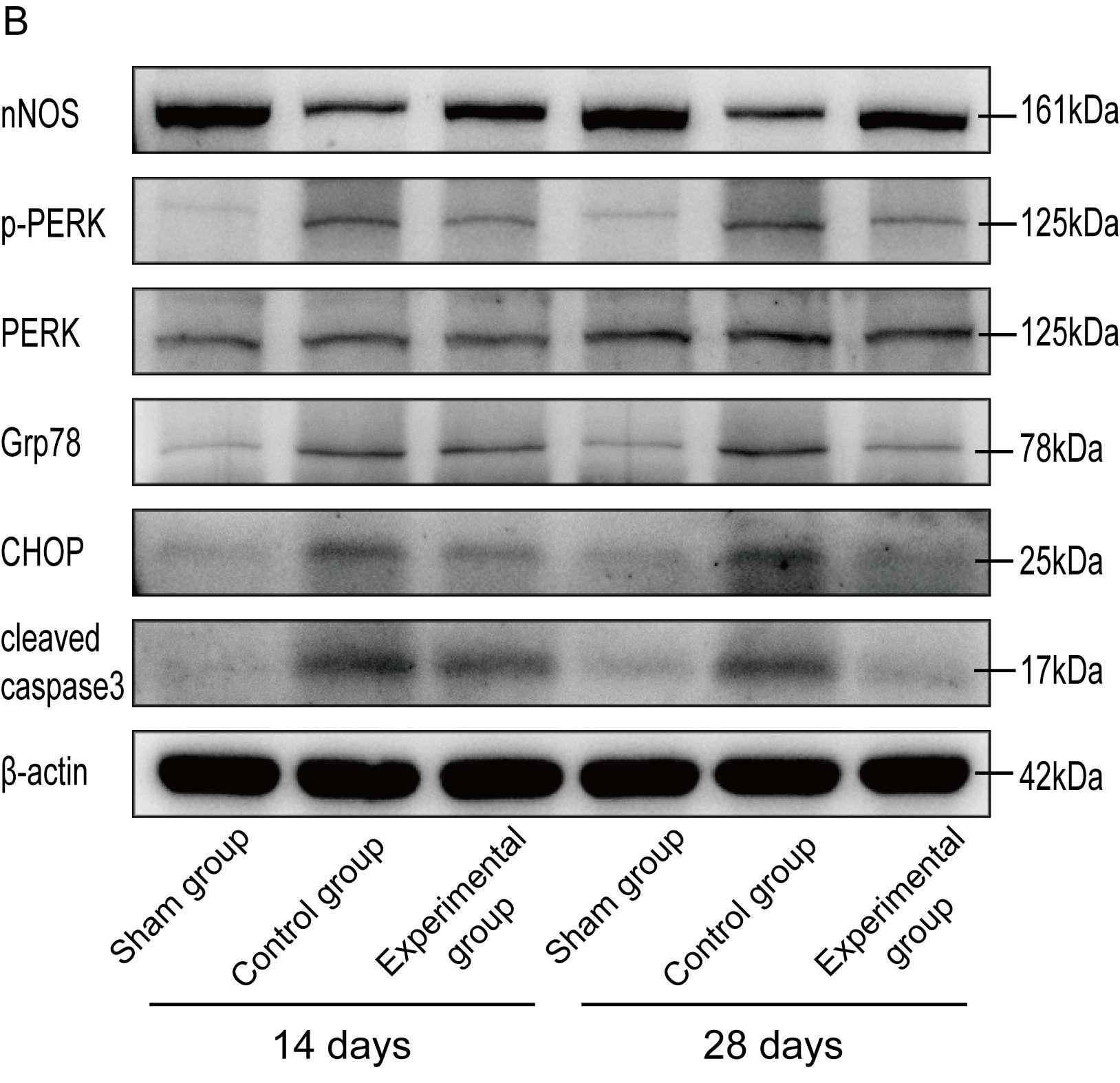


Sample 2

nNOS
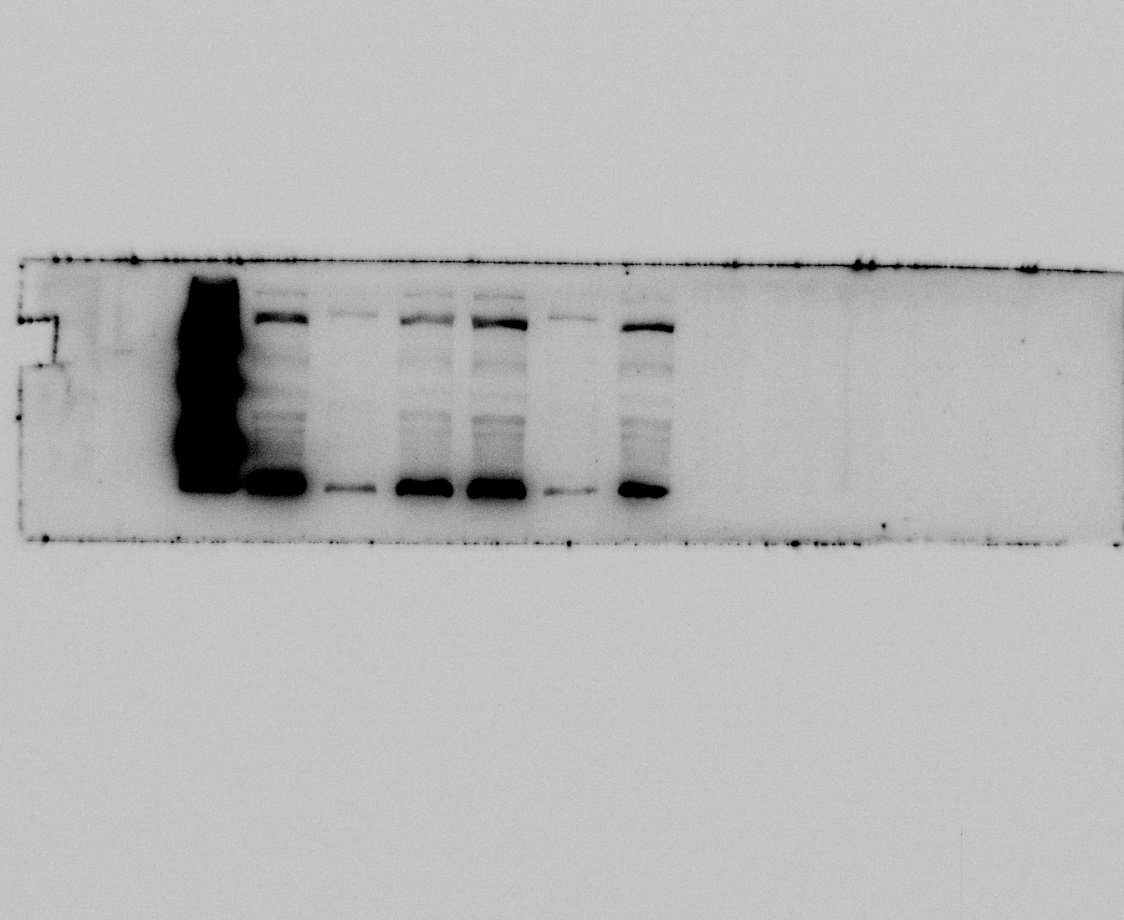


p-PERK
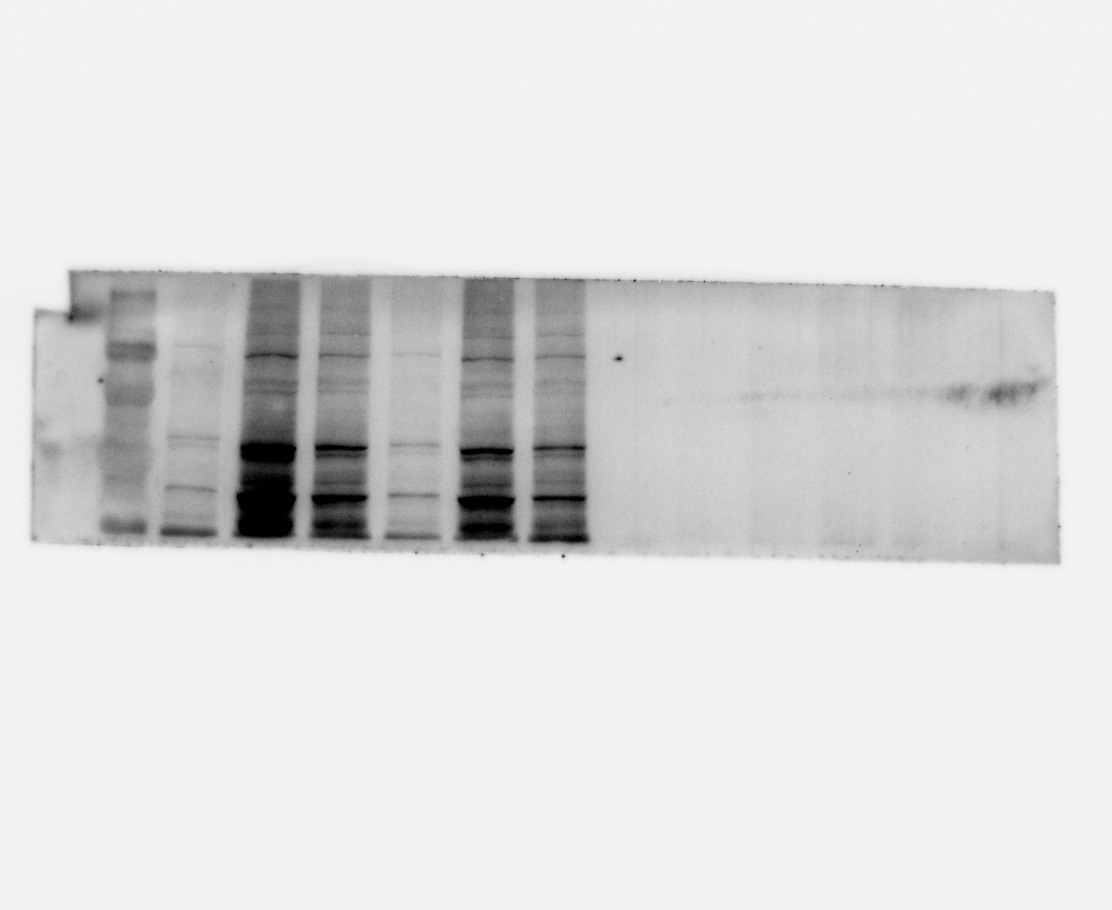


PERK
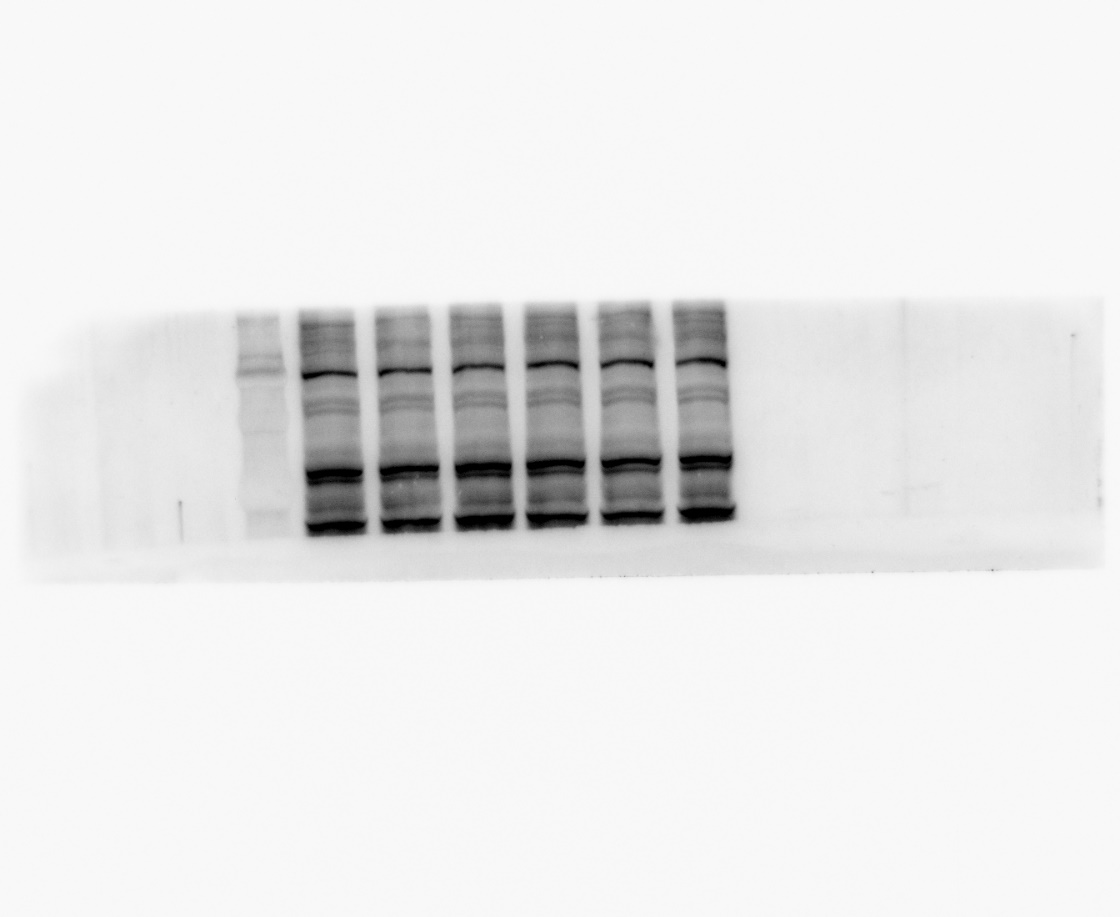


GRP78
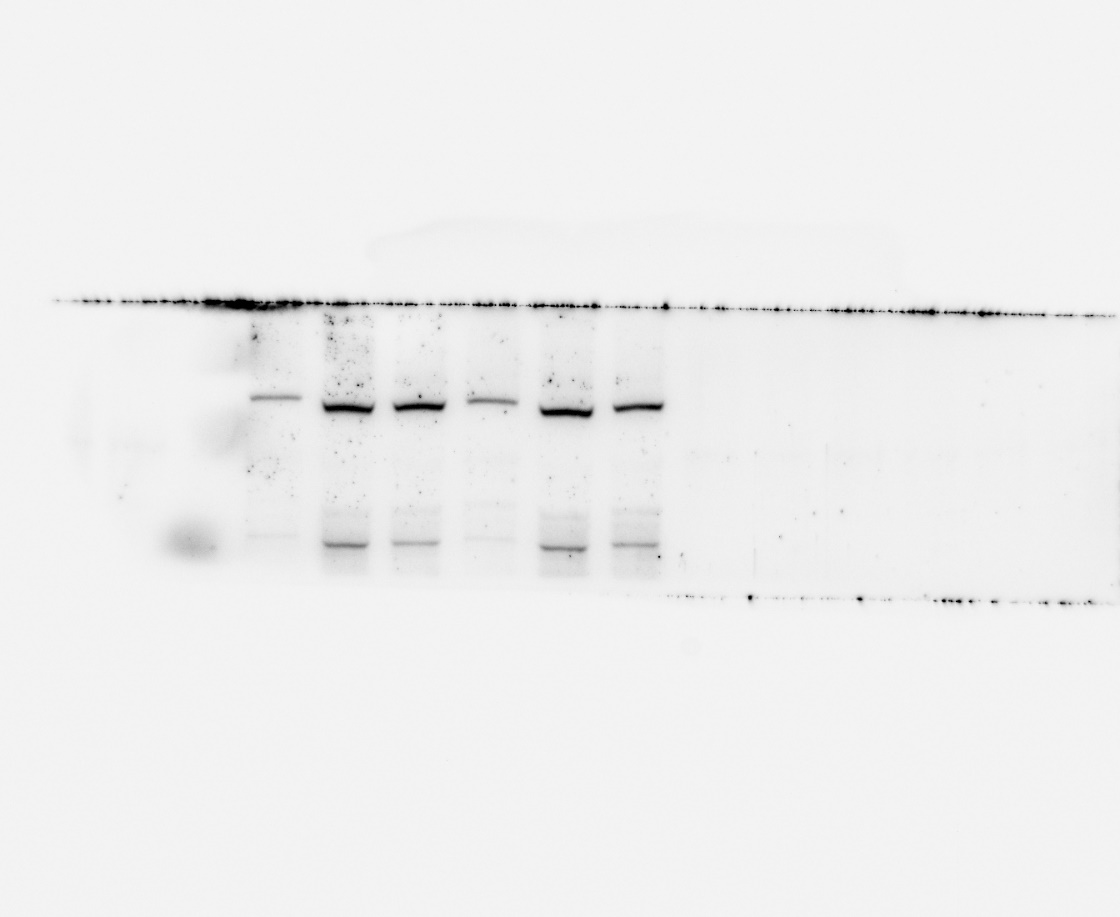


CHOP
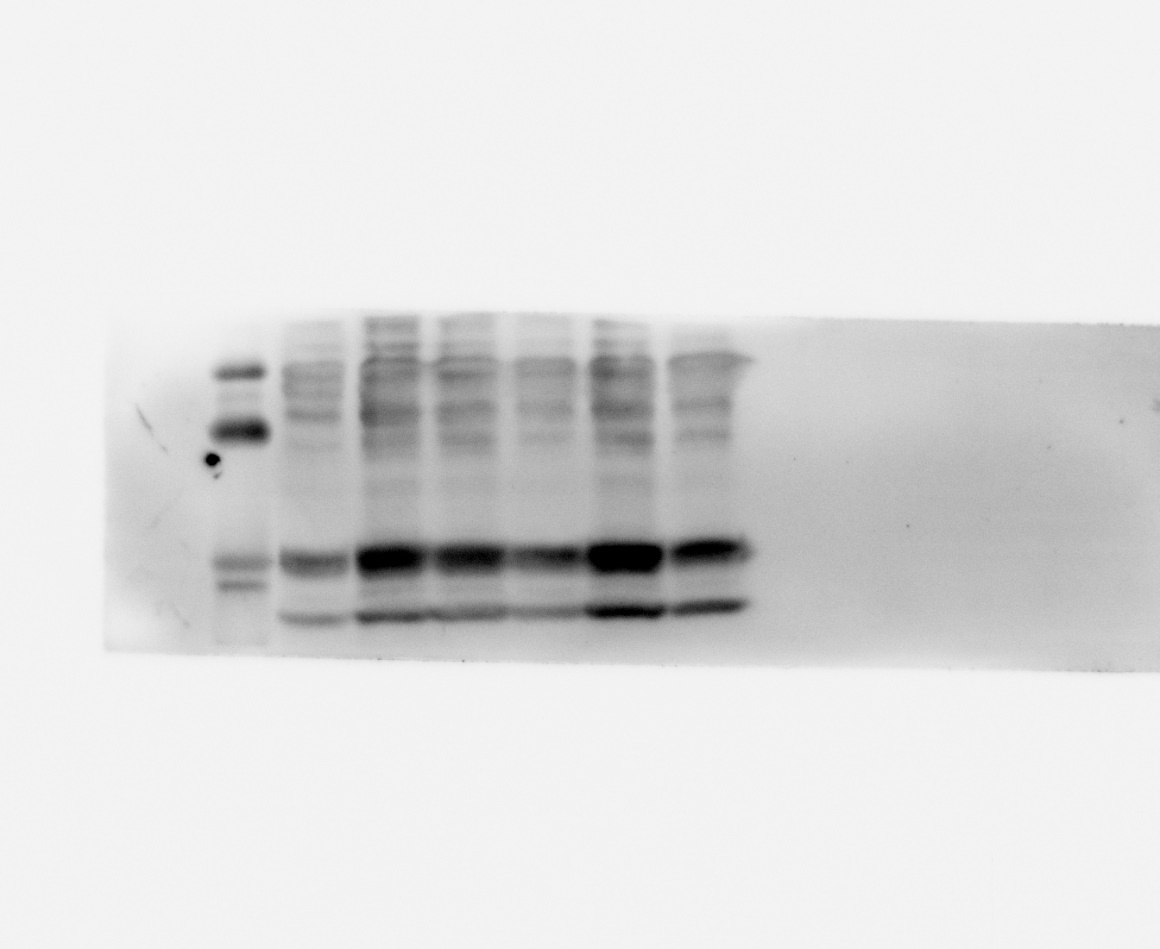


Cleaved caspase-3
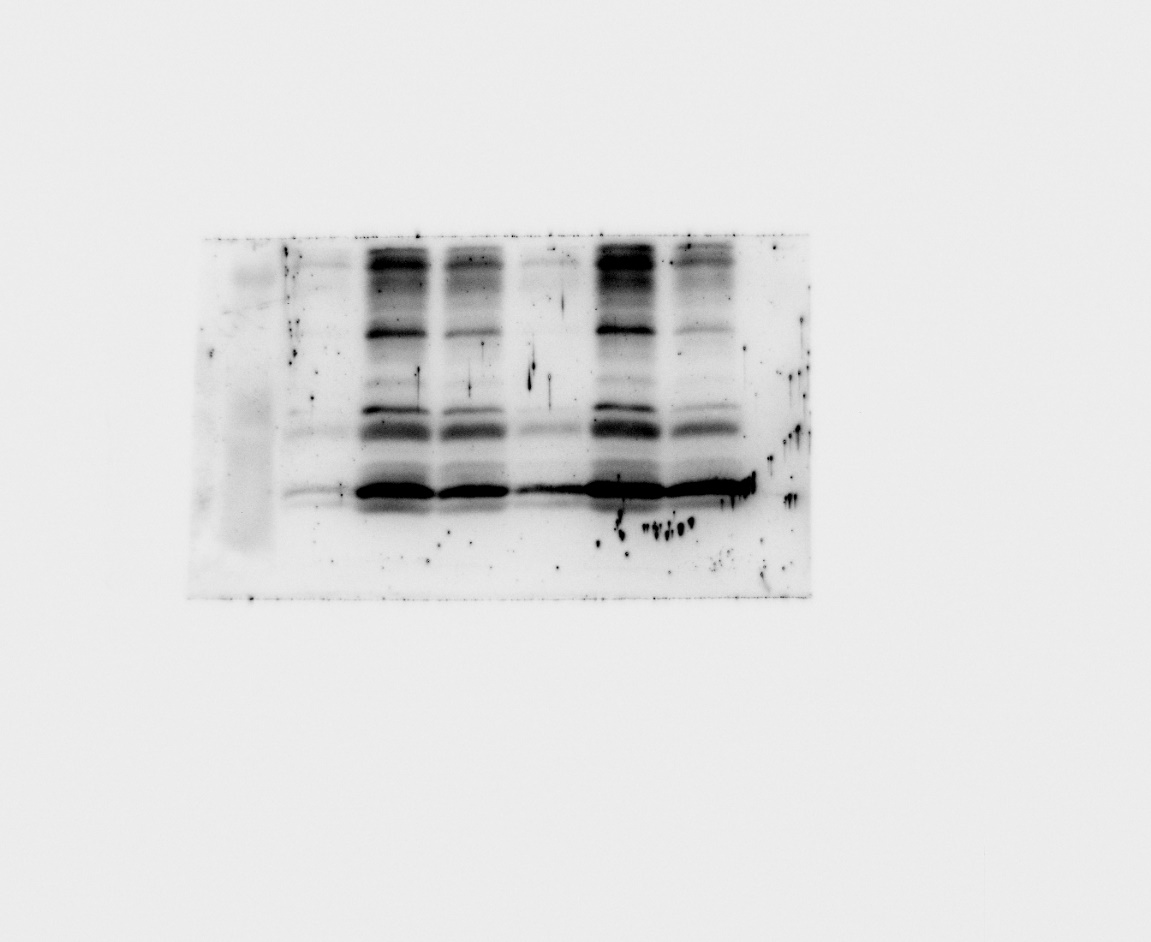


β-actin
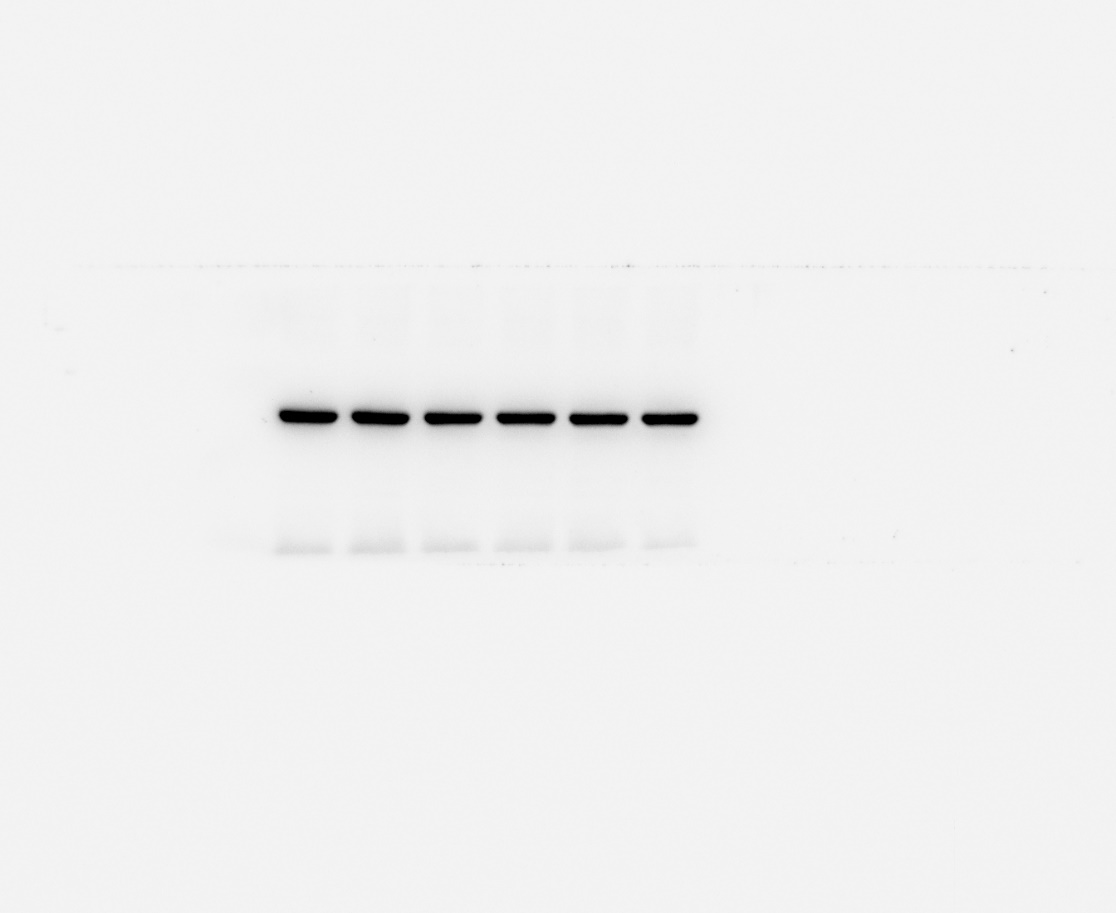


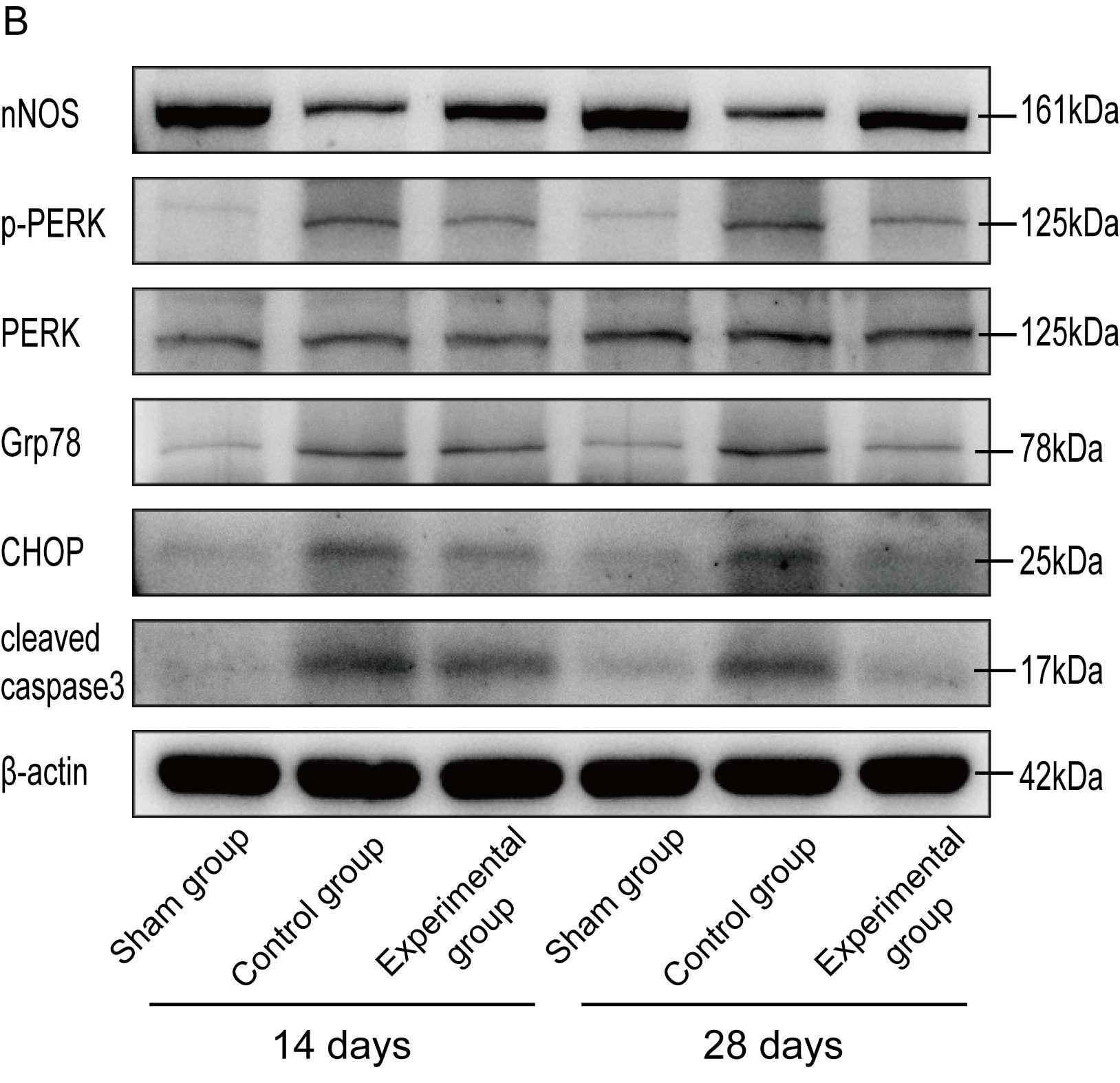


OPN
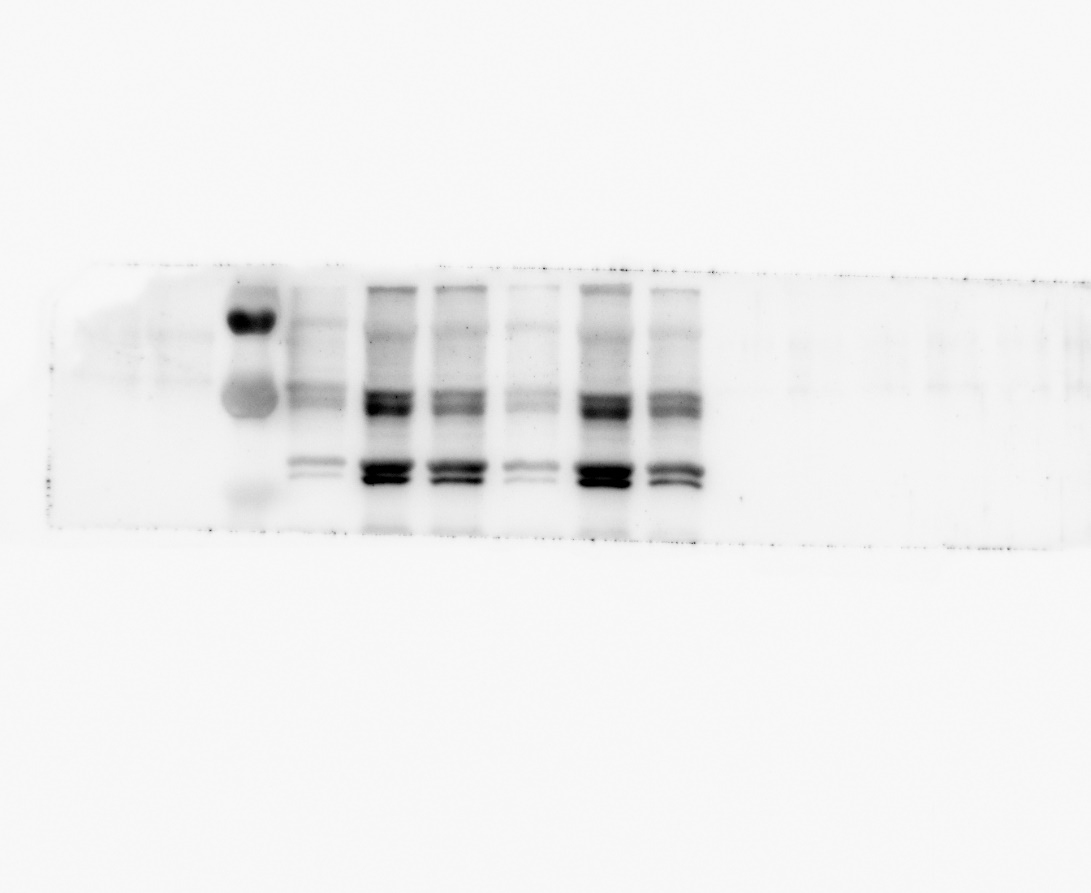


α-SMA
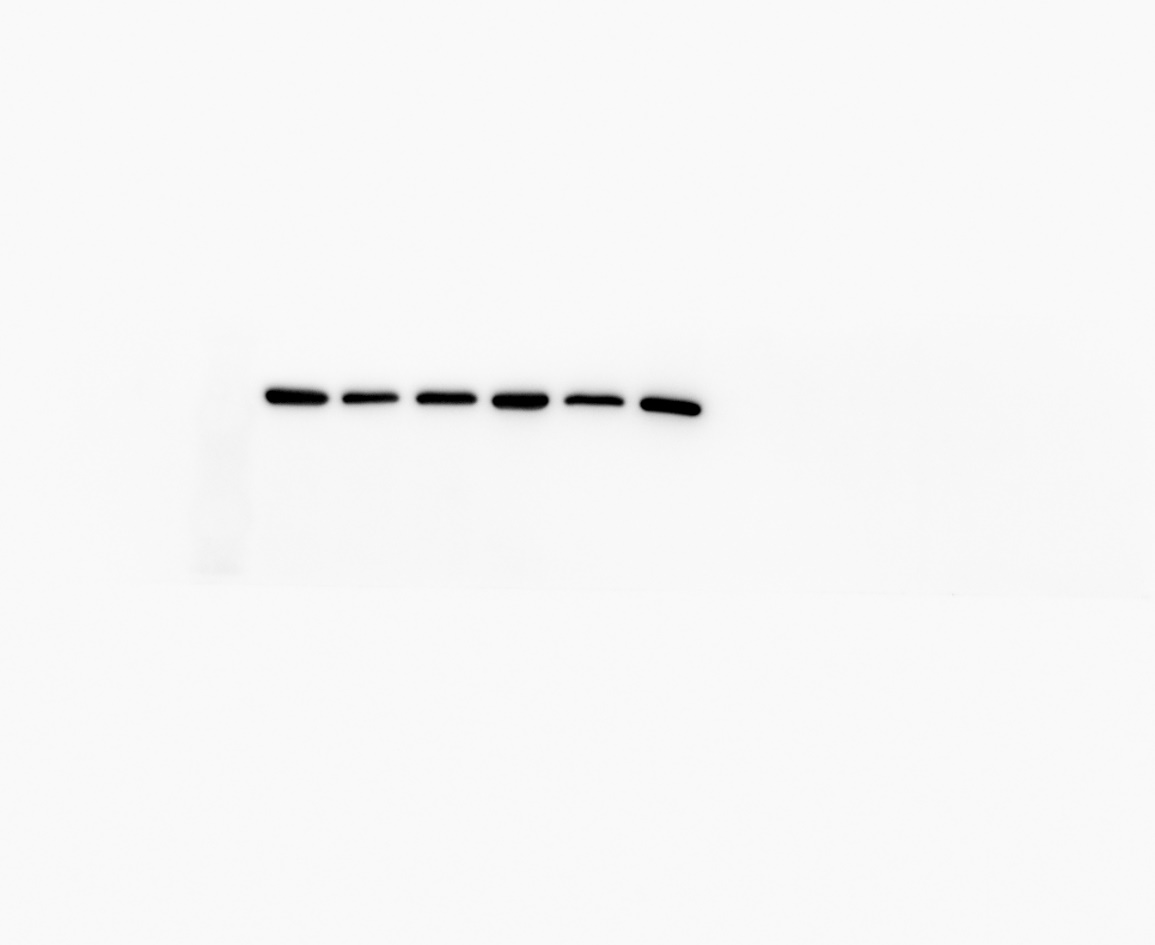


GAPDH
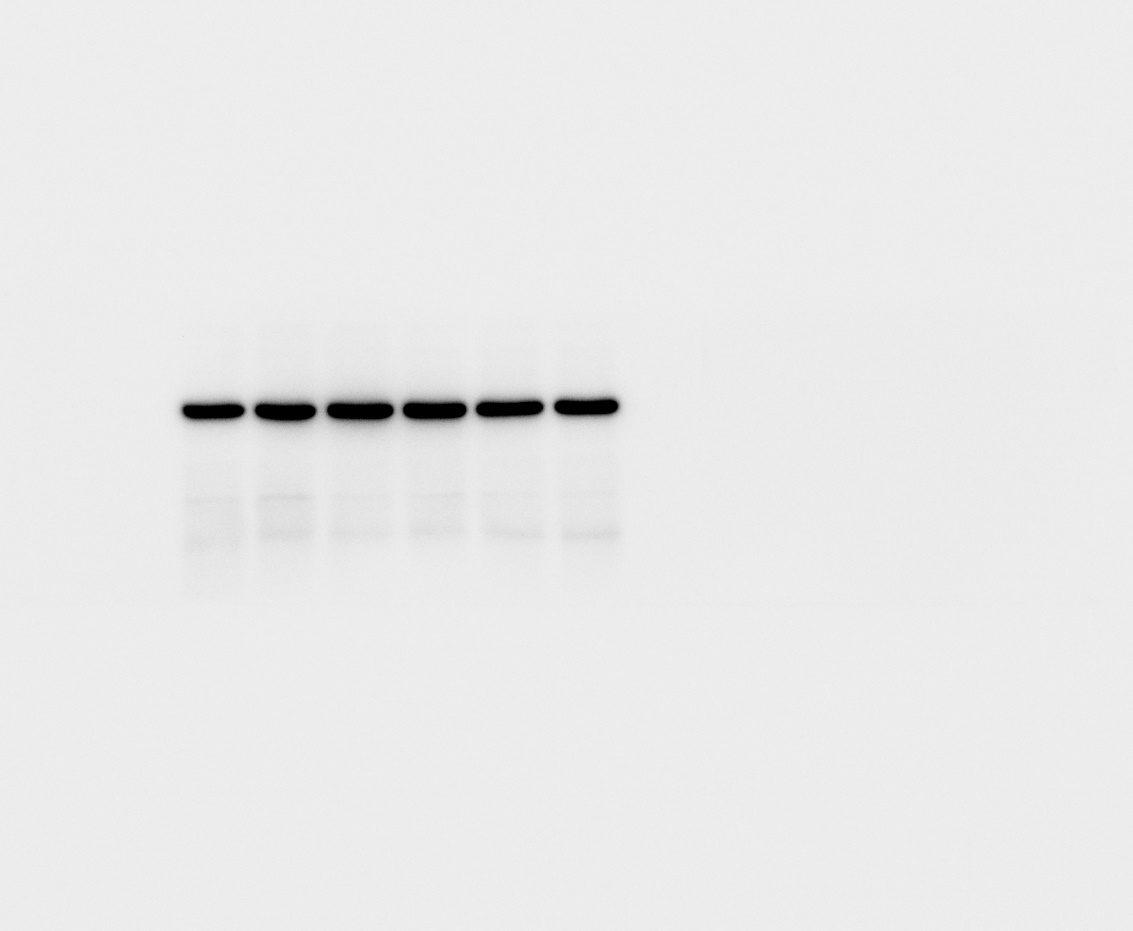


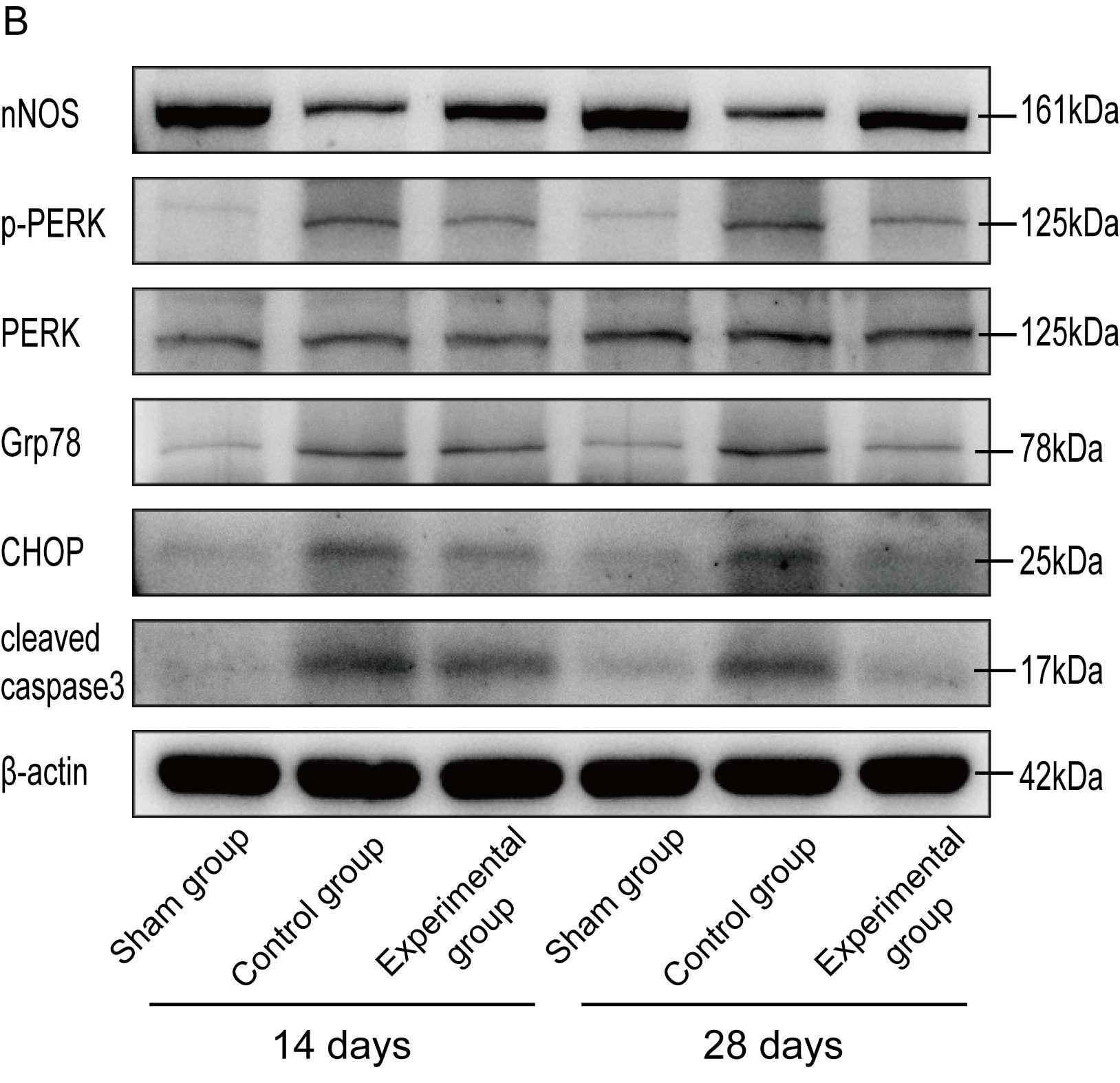


Sample 3

nNOS
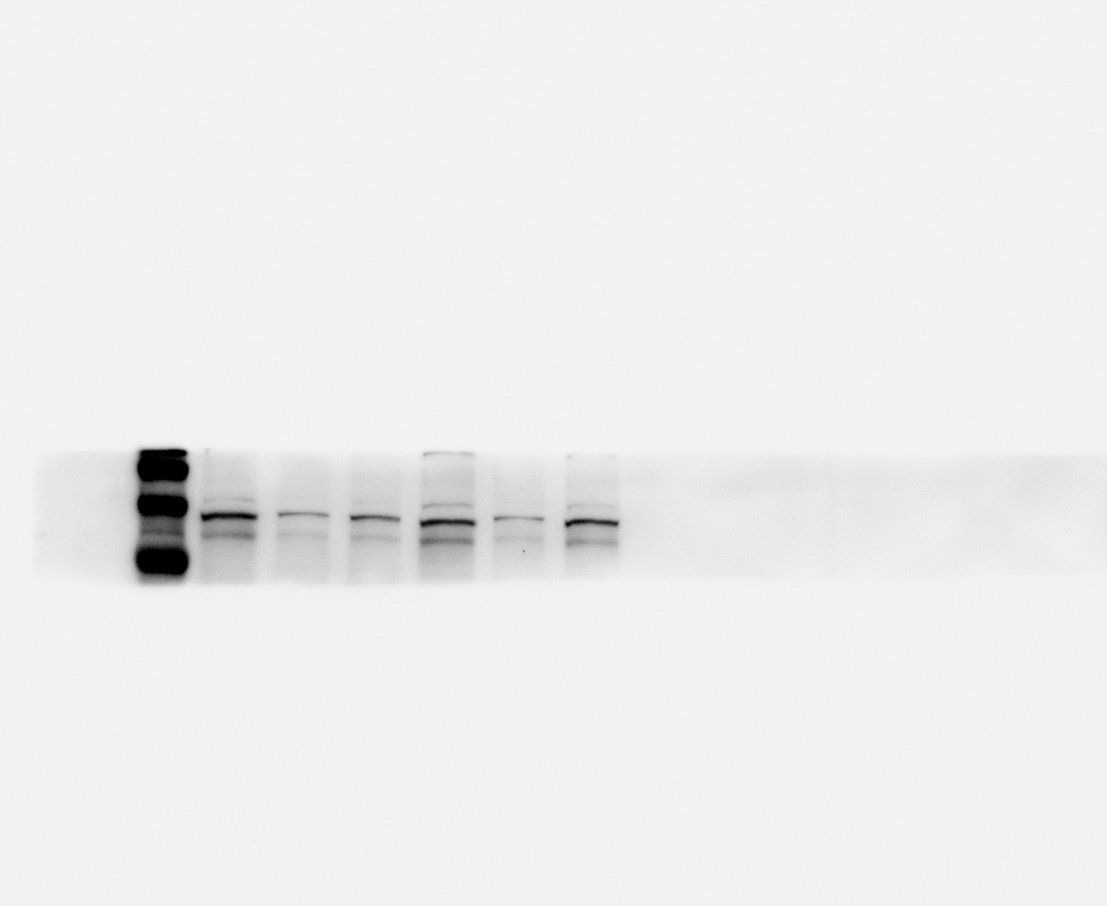


p-PERK
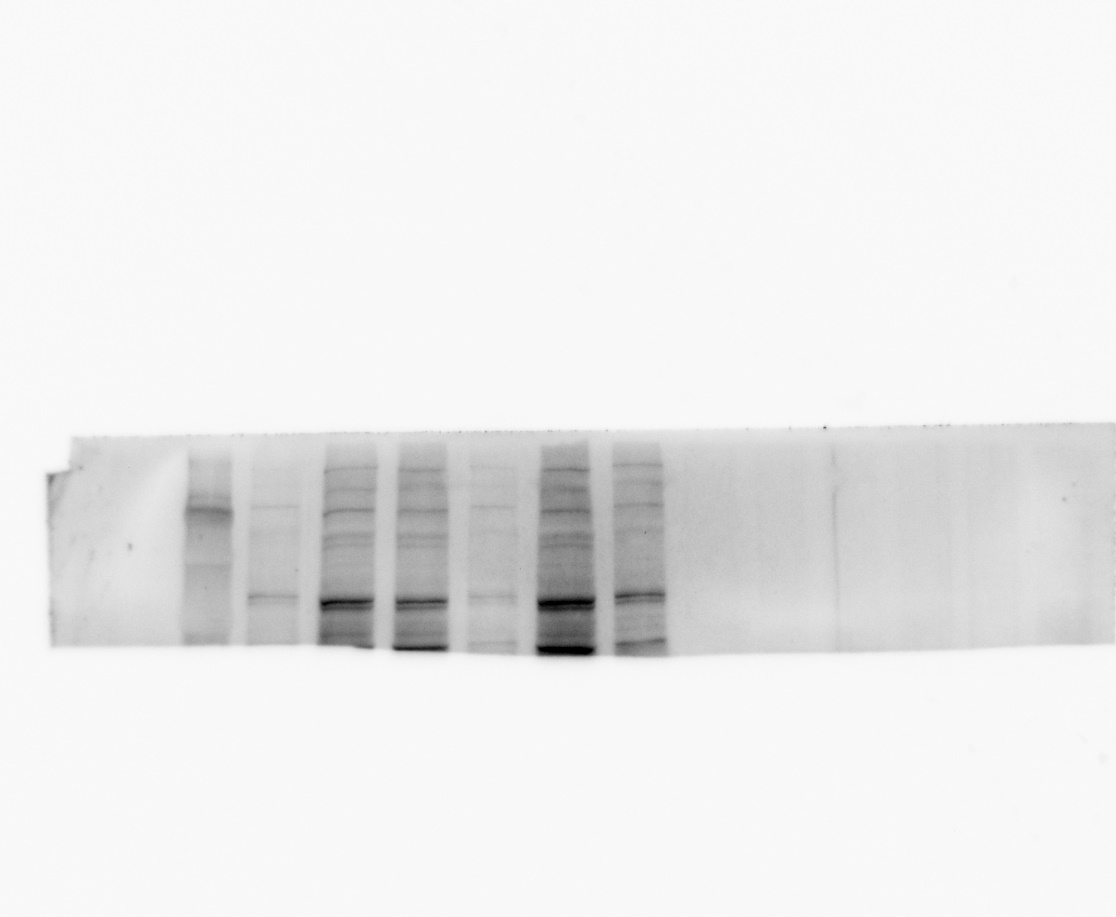


PERK
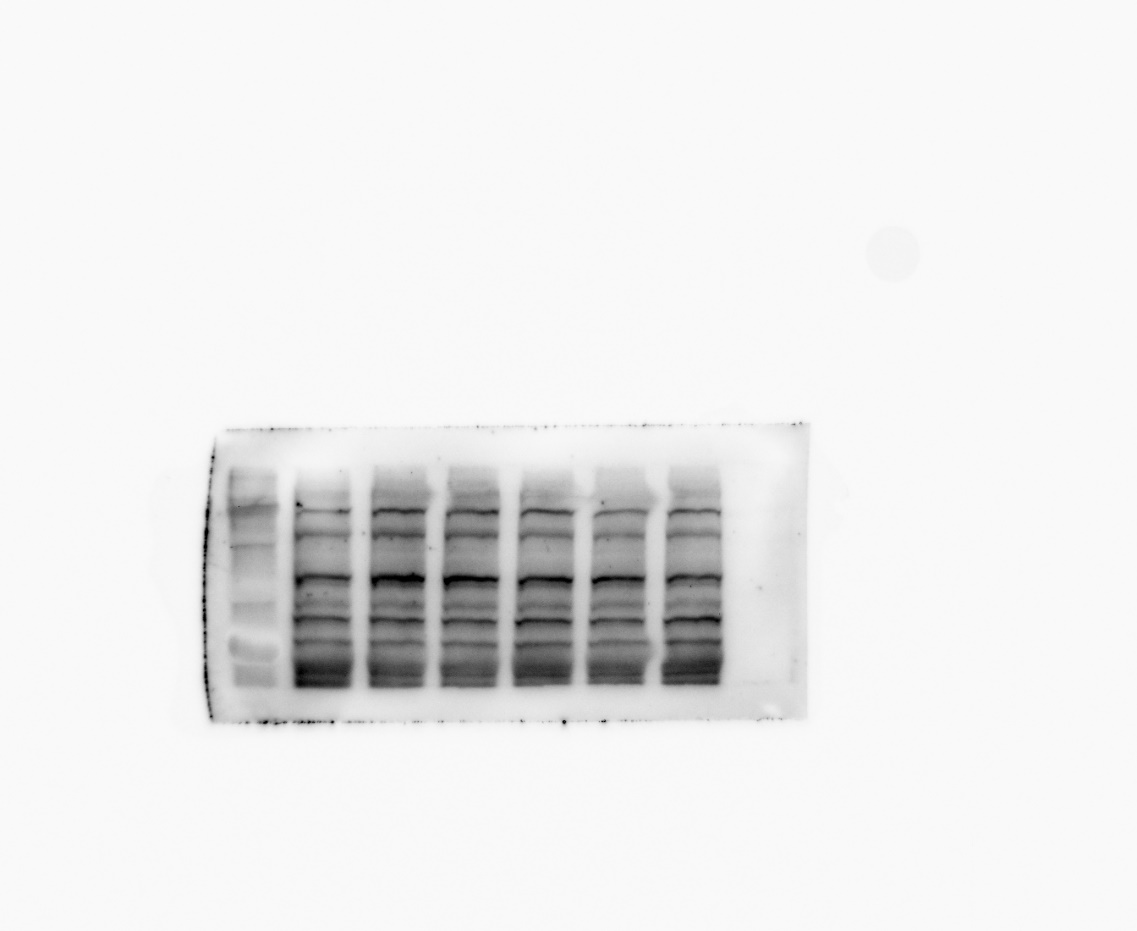


GRP78
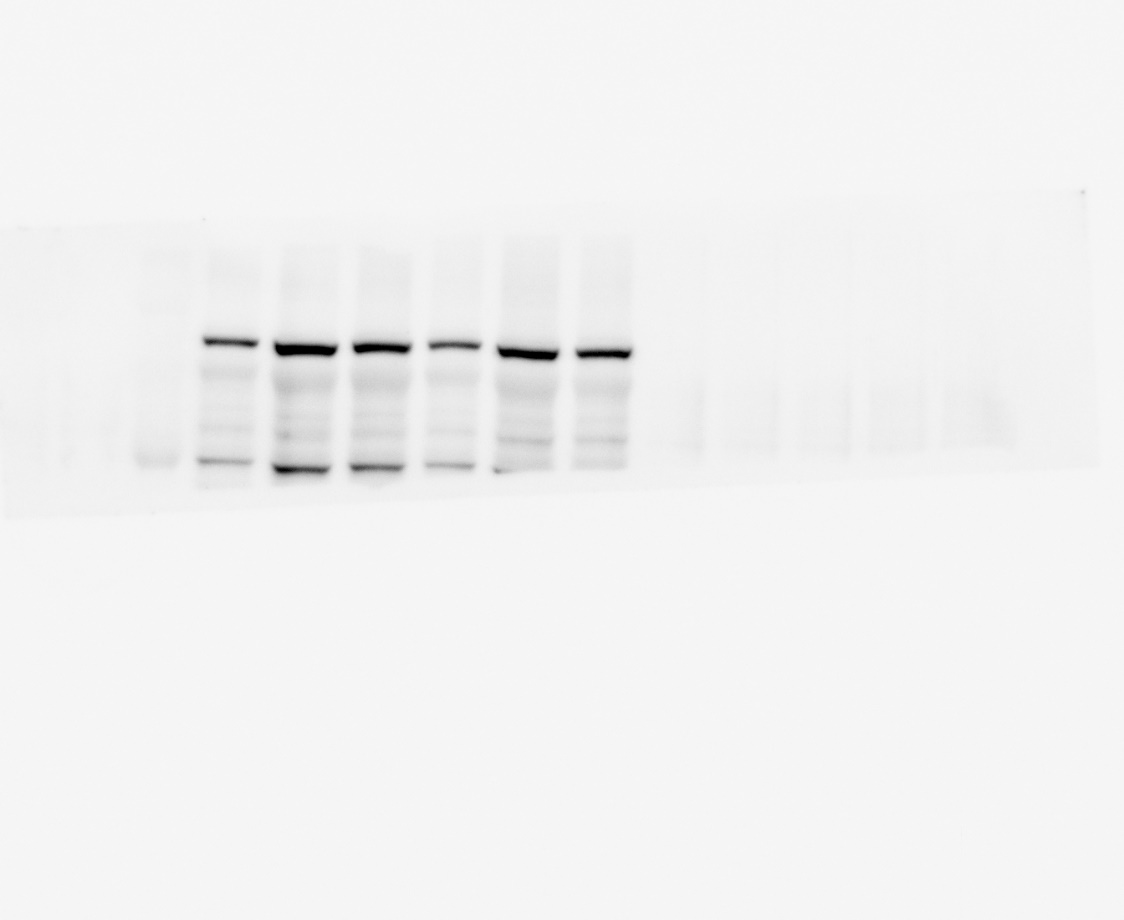


CHOP
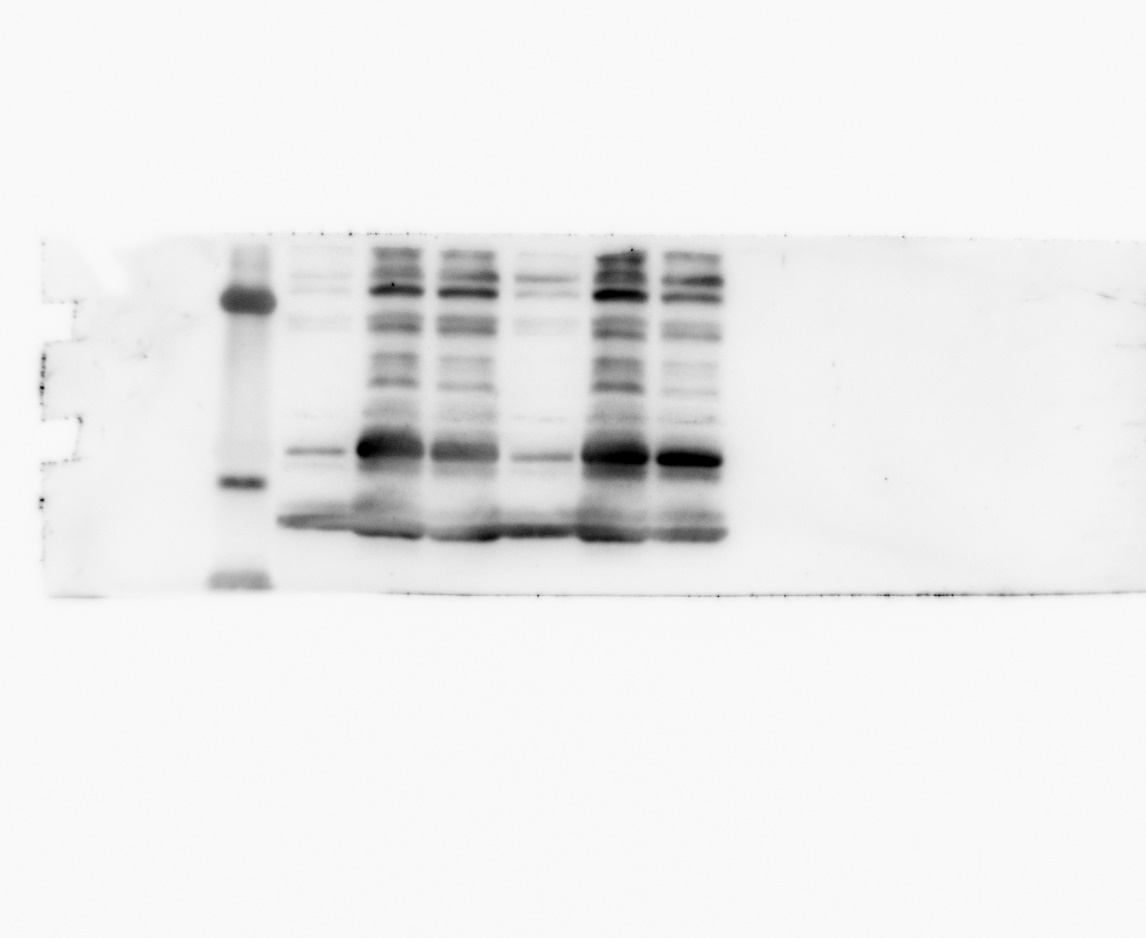


Cleaved caspase-3
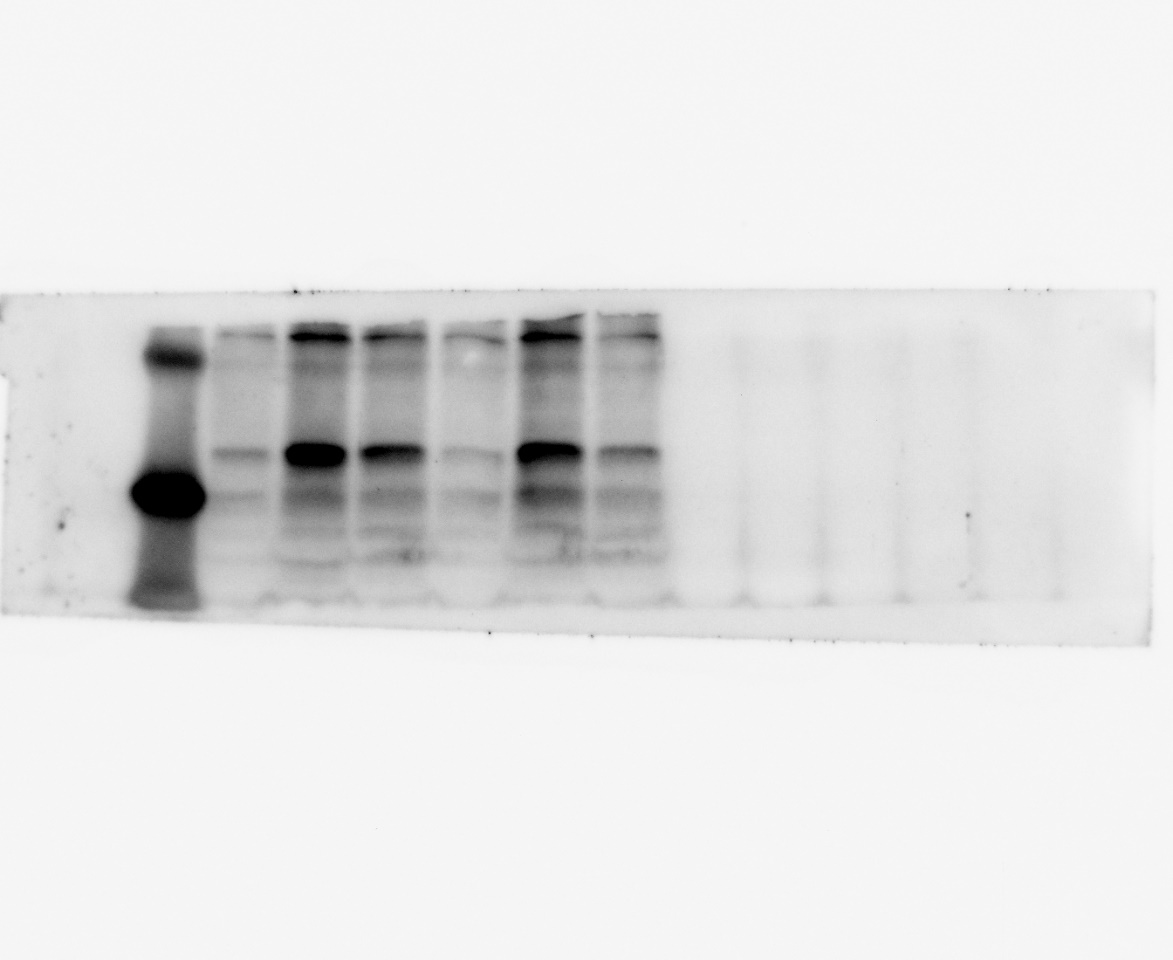


β-actin
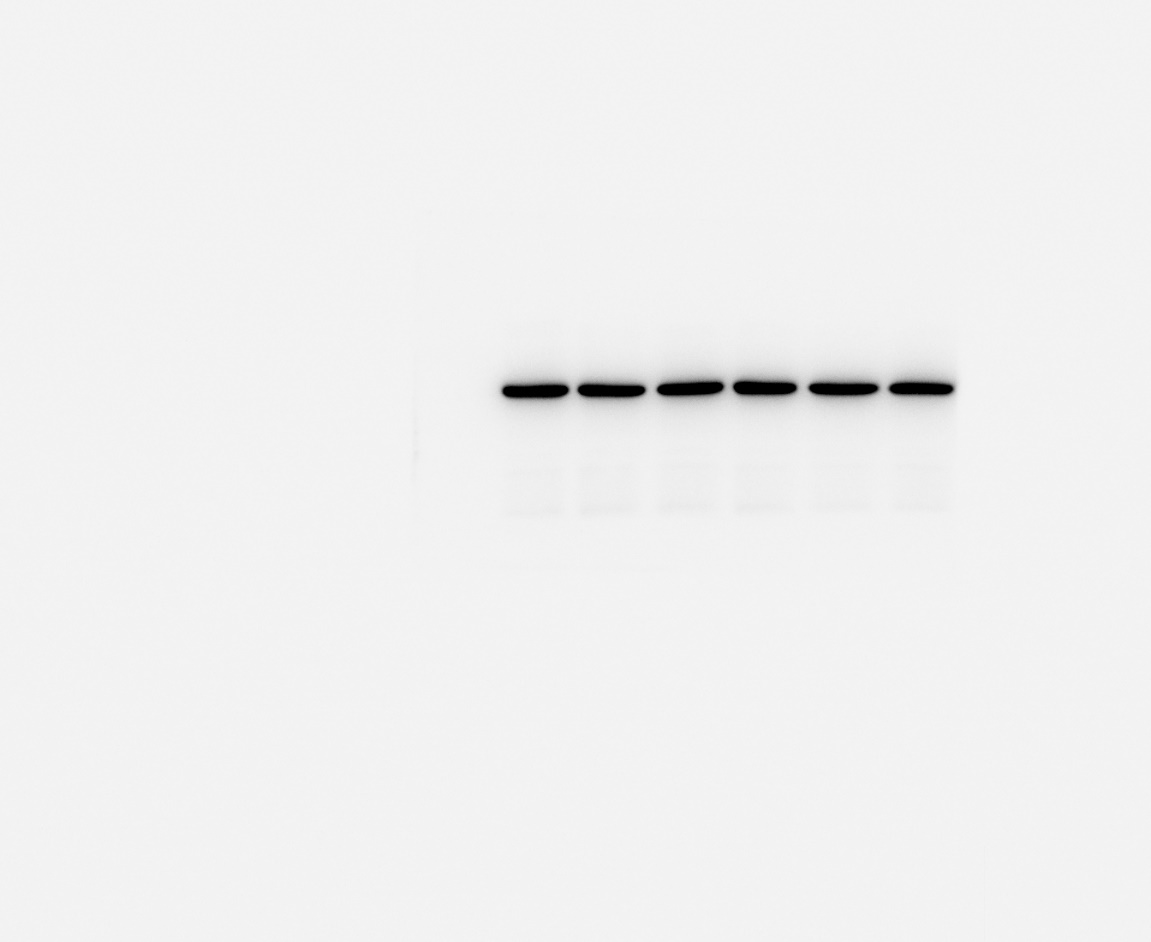


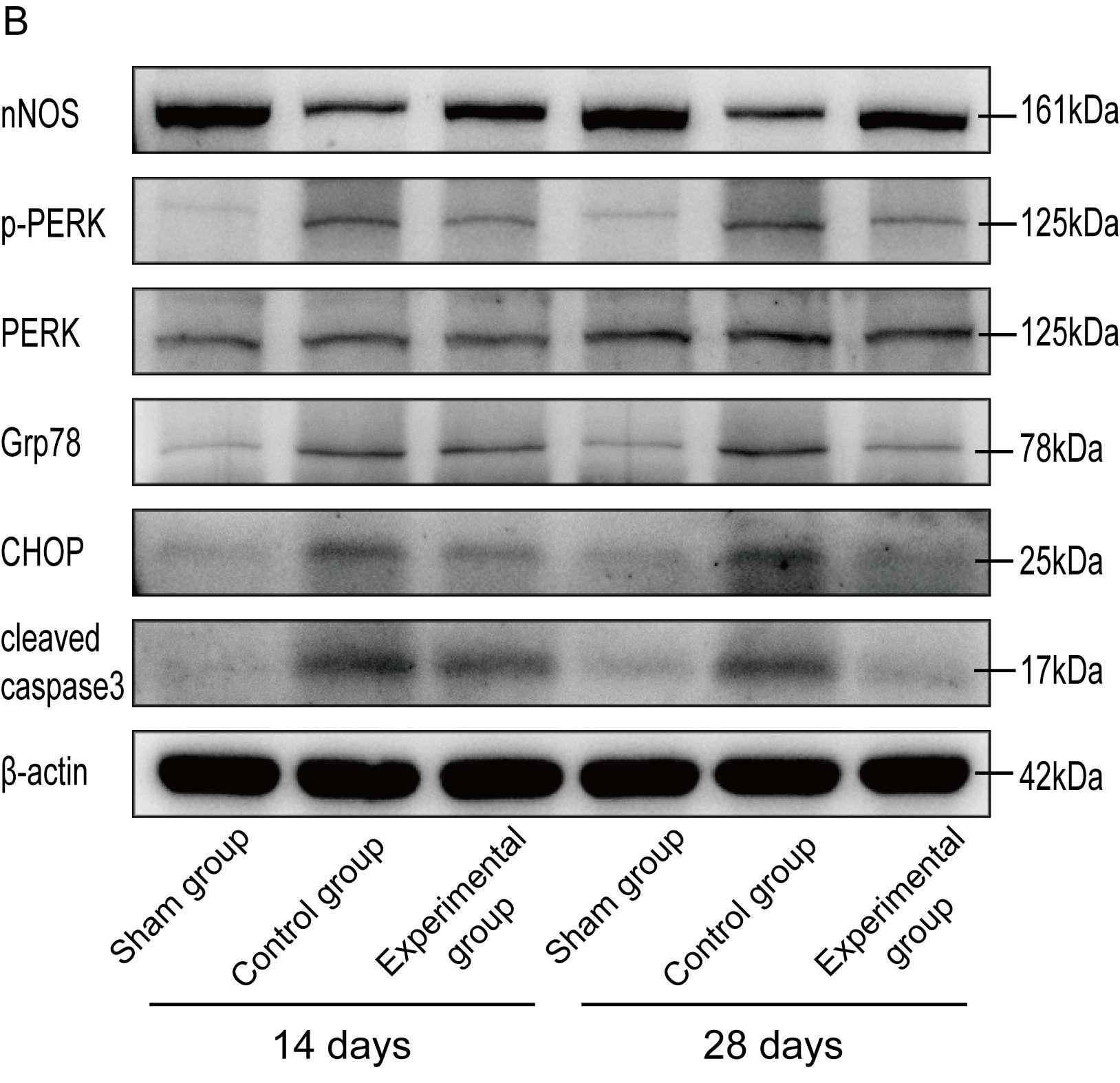


OPN
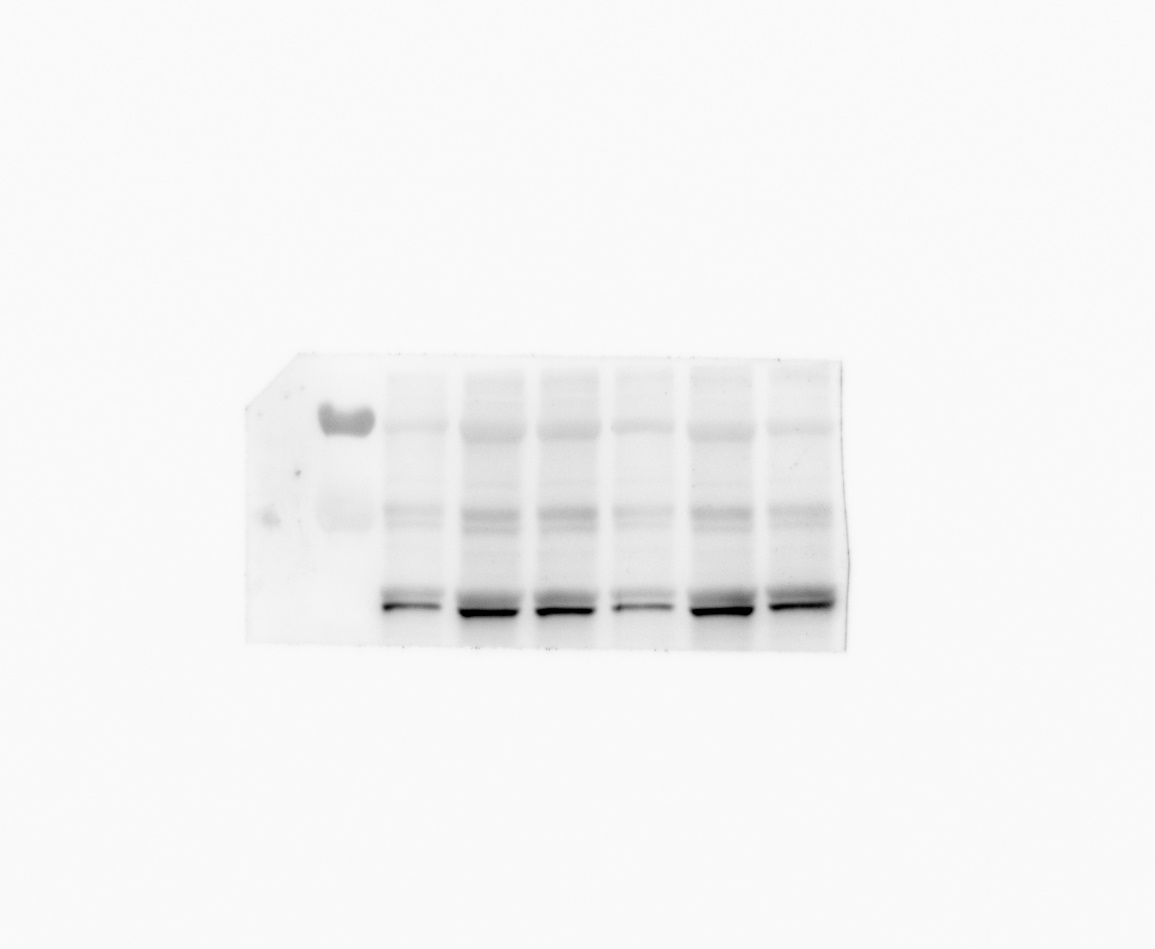


α-SMA
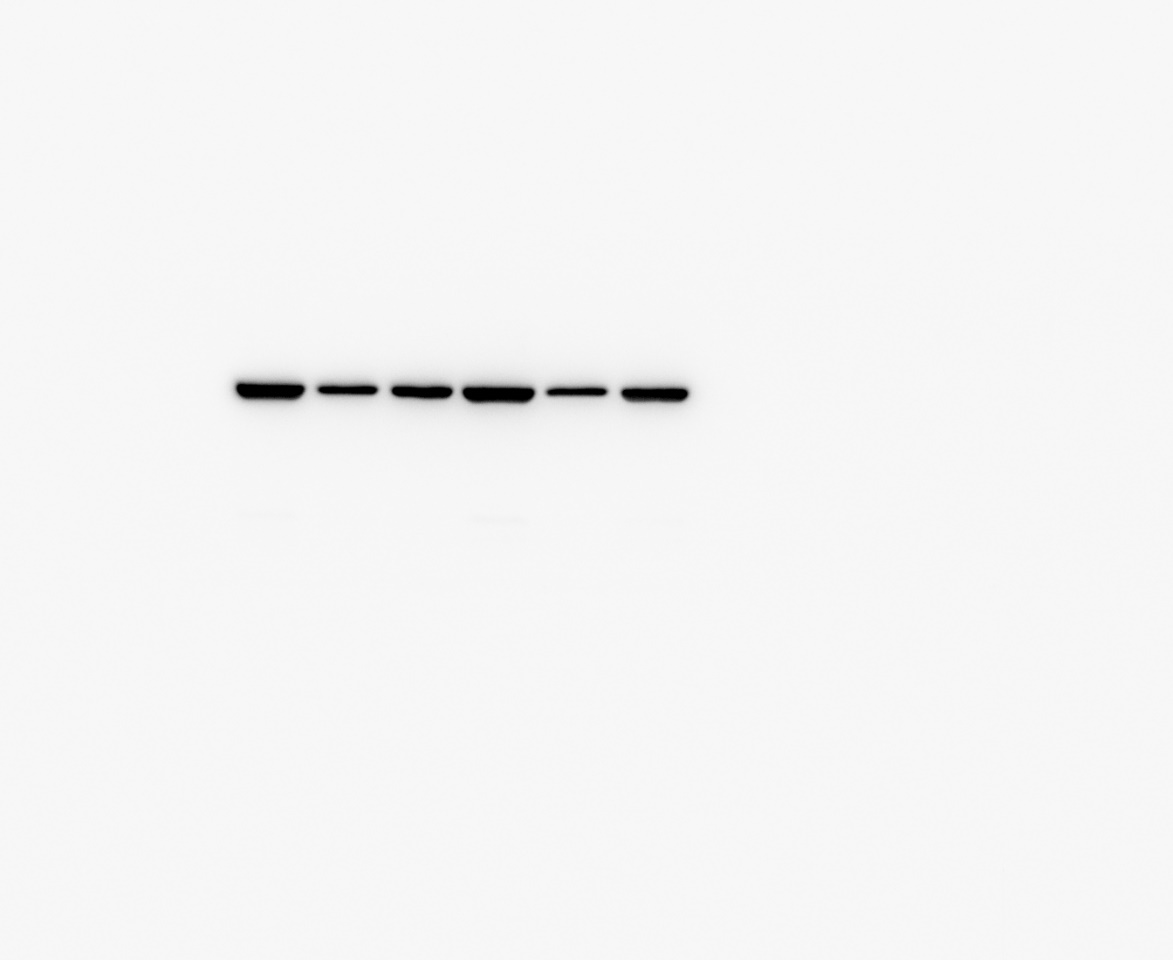


GAPDH
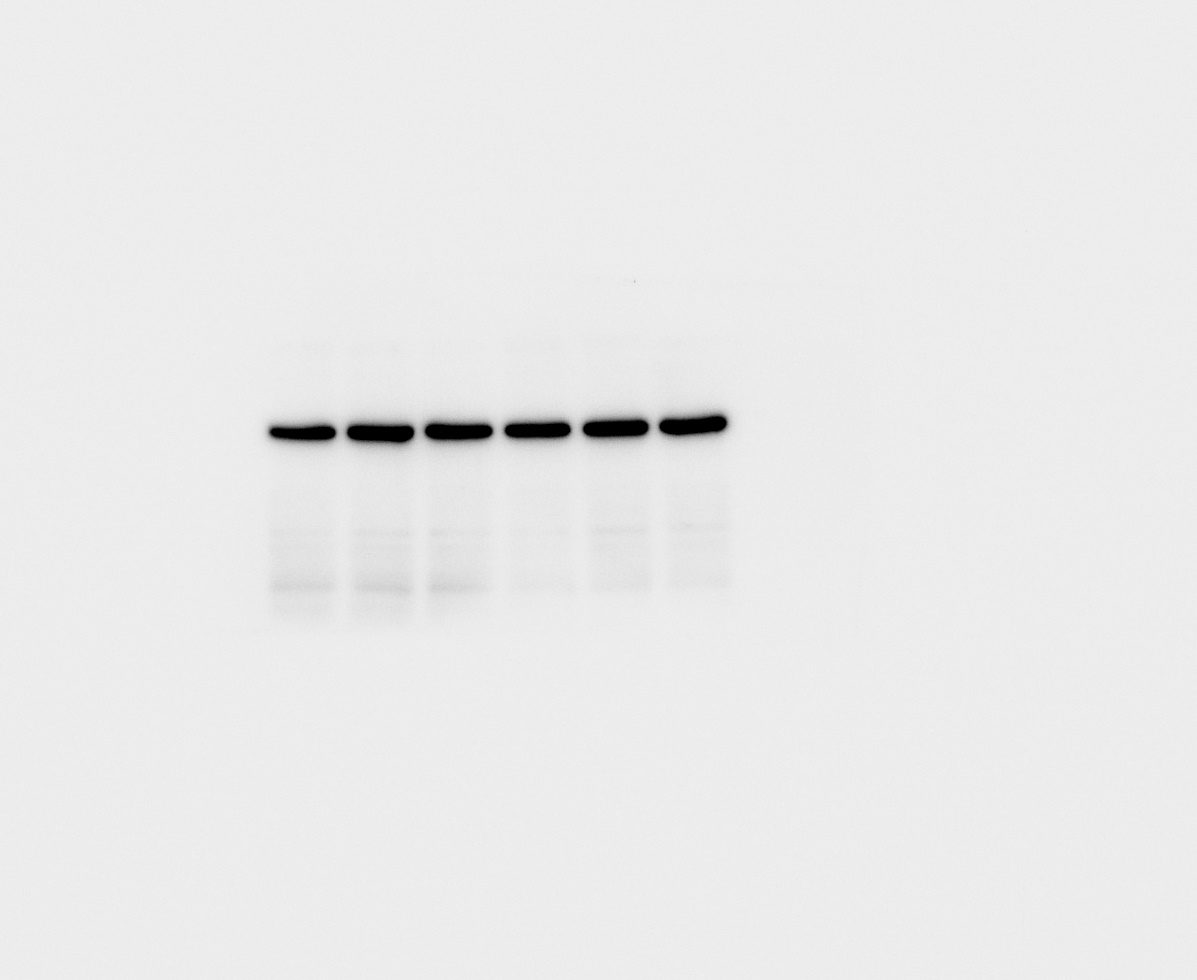


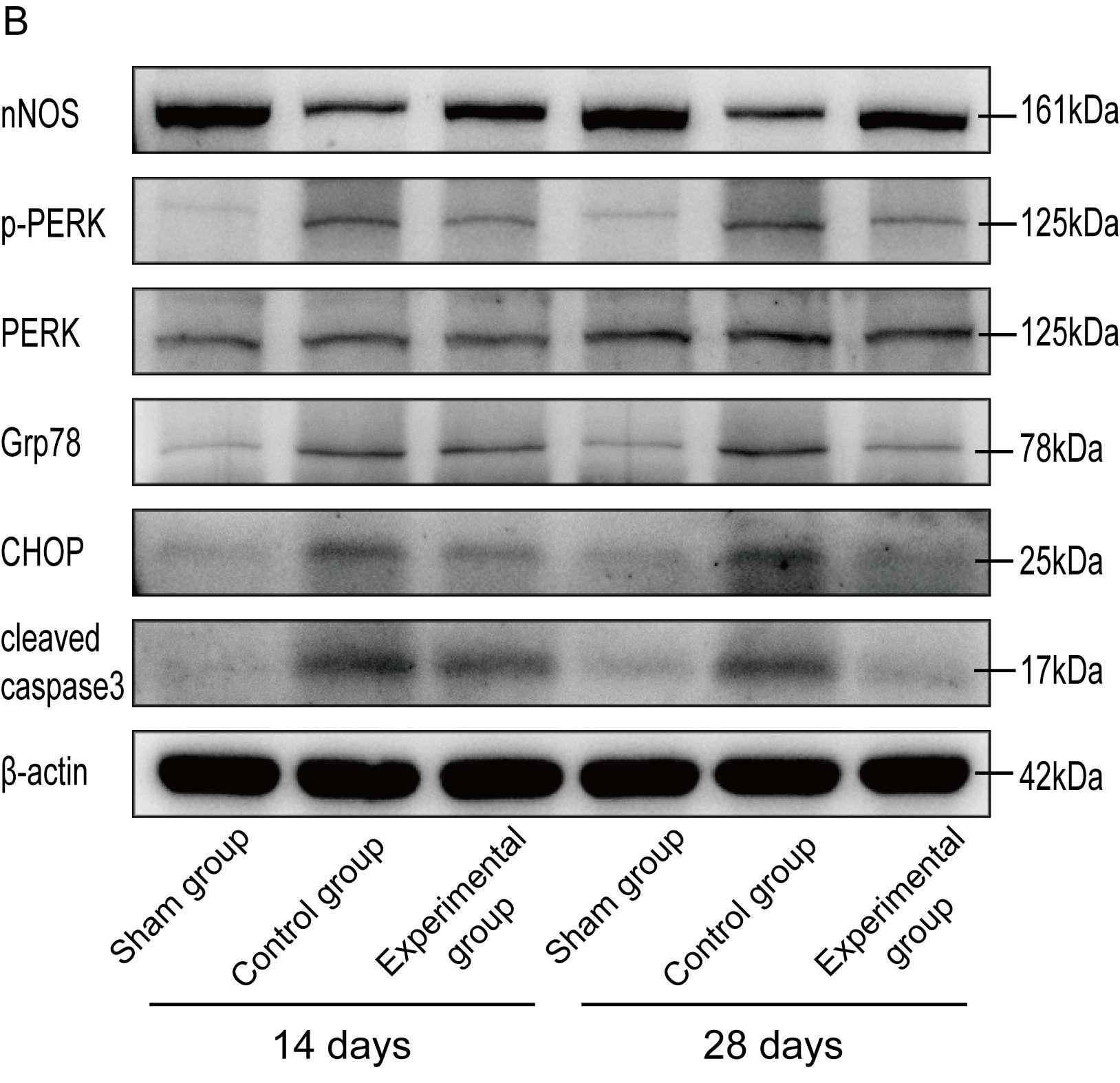

Supplement: WB_Supplemental_Figure1_qfad050 [file wb_supplemental_figure1_qfad050.doc]
