# Supplementary material for: Effects of endoplasmic reticulum stress on erectile function in rats with cavernous nerve injury
Source: Sex Med. 2023 Sep 3;11(4):qfad050. doi: 10.1093/sexmed/qfad050 (PMC10478027; doi:10.1093/sexmed/qfad050)
Supplement: Body_Weight_Supplemental_Table1_qfad050 [file body_weight_supplemental_table1_qfad050.doc]

Table 1. Body weights of each group

| Variable | Sham Group | Control group | Experimental group | P-value |
| --- | --- | --- | --- | --- |
| Initial weight (g)(n=12) | 327.75 ±5.99 | 328.50 ±6.88 | 327.40 ±5.87 | > 0.05 |
| Weight(days 14)(g)(n=6) | 405.15 ±10.95 | 402.45 ±9.52 | 401.87 ±9.29 | > 0.05 |
| Weight(days 14)(g)(n=6) | 526.63 ±12.65 | 524.65 ±10.25 | 524.60 ±10.47 | > 0.05 |

The data are presented as the mean ±SD.ANOVA and Bonferroni multiple comparison test were utilized to evaluate differences between groups.
